# MFPred: Rapid and accurate prediction of protein-peptide recognition multispecificity using self-consistent mean field theory

**DOI:** 10.1371/journal.pcbi.1005614

**Published:** 2017-06-26

**Authors:** Aliza B. Rubenstein, Manasi A. Pethe, Sagar D. Khare

**Affiliations:** 1 Computational Biology & Molecular Biophysics Program, Rutgers, The State University of New Jersey, Piscataway, NJ; 2 Institute for Quantitative Biomedicine, Rutgers, The State University of New Jersey, Piscataway, NJ; 3 Department of Chemistry and Chemical Biology, Rutgers, The State University of New Jersey, Piscataway, NJ; 4 Center for Integrative Proteomics Research, Rutgers, The State University of New Jersey, Piscataway, NJ; National Institutes of Health, UNITED STATES

## Abstract

Multispecificity–the ability of a single receptor protein molecule to interact with multiple substrates–is a hallmark of molecular recognition at protein-protein and protein-peptide interfaces, including enzyme-substrate complexes. The ability to perform structure-based prediction of multispecificity would aid in the identification of novel enzyme substrates, protein interaction partners, and enable design of novel enzymes targeted towards alternative substrates. The relatively slow speed of current biophysical, structure-based methods limits their use for prediction and, especially, design of multispecificity. Here, we develop a rapid, flexible-backbone self-consistent mean field theory-based technique, MFPred, for multispecificity modeling at protein-peptide interfaces. We benchmark our method by predicting experimentally determined peptide specificity profiles for a range of receptors: protease and kinase enzymes, and protein recognition modules including SH2, SH3, MHC Class I and PDZ domains. We observe robust recapitulation of known specificities for all receptor-peptide complexes, and comparison with other methods shows that MFPred results in equivalent or better prediction accuracy with a ~10-1000-fold decrease in computational expense. We find that modeling bound peptide backbone flexibility is key to the observed accuracy of the method. We used MFPred for predicting with high accuracy the impact of receptor-side mutations on experimentally determined multispecificity of a protease enzyme. Our approach should enable the design of a wide range of altered receptor proteins with programmed multispecificities.

## Author summary

Across biology, many proteins that recognize peptides are multispecific; they interact with multiple binding partners of disparate sequence. Computational prediction of these multiple peptide partners would enable greater understanding of individual protein-recognition domains. Additionally, the ability to customize protein-recognition domains by designing them to recognize and act upon a new set of peptides and not bind their original binding partners would be useful in drug design and biotechnology. Current methods for predicting multispecificity operate on a timescale that is too slow to be used for design. Here, we present a method, MFPred, for predicting multispecificity. MFPred robustly recapitulates protein-recognition domain specificity for a range of proteins, at comparable accuracy and with considerable speed-up relative to current methods. We apply MFPred to predicting altered multispecificity in a mutant protease to demonstrate its relevance to design. The rapidity and accuracy of MFPred should enable its use in investigating and modulating biological processes.

## Introduction

Many natural proteins, including signal transduction hubs and enzymes that process biological information, have evolved to be multispecific–they participate in specific interactions with several interaction partners [1,2]. Evolution of multispecificity includes selection for both positive and negative specificity, involving recognition and non-recognition, respectively, of sets of interaction partners [3]. Most multispecific interactions arise when the active site of a single receptor protein interacts with multiple binding partners of differing sequence [4]. Nature uses structurally conserved protein-recognition domains (PRDs), e.g., SH2, SH3 and PDZ domains, to mediate many multispecific interactions [5–10]. Thus, it is crucial that methods that model and modulate PRD specificity are able to accurately recapitulate their multispecific nature.

Similar to cascades composed of multispecific PRDs like SH3, SH2 and PDZ domains that mediate signal transduction, proteolytic cascades are ubiquitous in the post-translational transduction of biological information [11]. Protease activity and selectivity is involved in a diverse range of biological processes including digestion, blood clotting, apoptosis and cancer [12–15]. Proteases are inherently multispecific such that they recognize and proteolyze (or cleave) a range of substrates (positive specificity) while not recognizing others (negative specificity) [3]. For example, viral proteases such as HCV protease that are involved in viral maturation cleave only specific sites in the viral polyprotein but do not cleave others [16]. These proteases may also have evolved the ability to cleave specific host proteins [17]. Prediction of protease multispecificity is, therefore, key for identifying their substrates under healthy and disease conditions. Additionally, designed proteases with programmed multispecificity have the potential to be used as therapeutics and protein-level knockout reagents in cell culture [18]. The ability to manipulate protease specificity computationally would enable the creation of such designer proteases with dialed-in recognition specificity, thereby providing tools to interrogate and intervene in biological processes.

Rational modulation of protein-protein or protein-peptide interaction multispecificity has met with limited success, except in a few notable cases, such as coiled-coil interfaces [19,20]. In principle, computational structure-based modeling methods should be able to recapitulate and modulate multispecificity. In fact, several methods relying on, among others, Monte-Carlo (MC) simulations in sequence and conformation space, and genetic algorithms (GA) have been developed to predict PRD multispecificity [21–25]. However, these methods are limited by the time required to enumerate a sufficiently large number of sequences to sample the substrate/peptide sequence space. As multispecific design entails additional sampling of (thousands) of receptor variants and modeling the multispecificity of each variant separately, using current methods to design receptors for and against specificity profiles is not computationally feasible.

We have developed a structure-based method that eliminates the expense of explicit sequence enumeration in multispecificity modeling. The method uses a self-consistent **M**ean-**F**ield theory-based **Pred**iction (MFPred) approach that expresses specificity as a sitewise probability distribution function that can be calculated relatively rapidly. We have benchmarked MFPred on four diverse proteases and compared the results to MC- and GA-based methods. MFPred has comparable accuracy to MC-based and GA-based methods and provides a tens- to thousands-fold speedup. We demonstrate the generality of MFPred by obtaining significant multispecificity predictions for five diverse classes of protein-recognition domains (PRDs). Finally, as a proof-of-concept for design, we demonstrate that MFPred can recapitulate experimentally determined changes in specificity profiles due to receptor-side mutations.

## Results

### Self-consistent mean field theory-based specificity profile prediction algorithm

**Fig 1:**
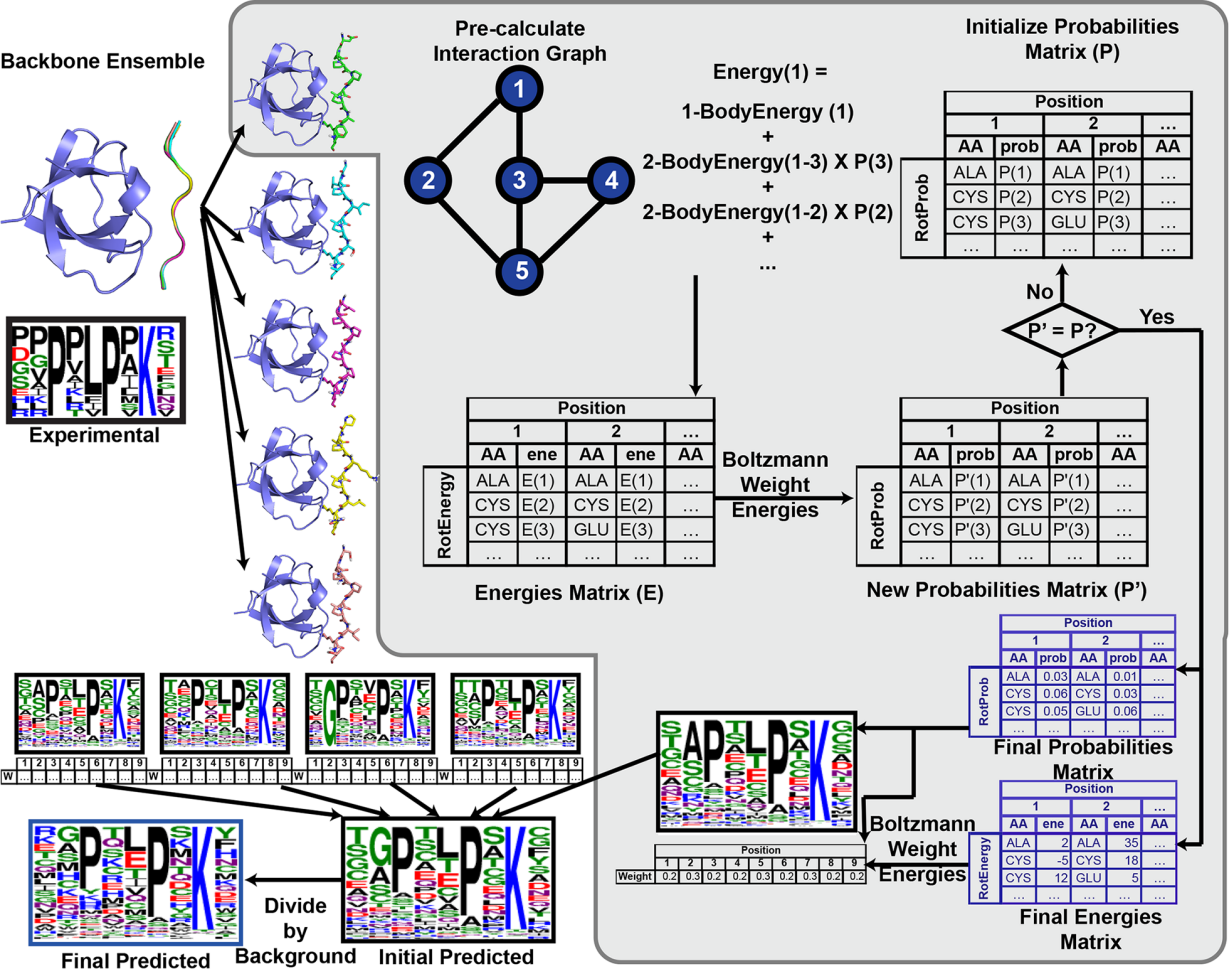
MFPred workflow. MFPred input is a backbone ensemble of a protein/peptide complex, which is generated from a protein structure from the PDB (1CKA here) as described in Methods. For each backbone, Rosetta pre-calculates the interaction graph, which stores intrinsic rotamer one-body energies on the vertices (blue circles) and matrices of rotamer-rotamer two-body energies on the edges (black lines). A probabilities matrix (P) is initialized. Mean-field energies (E) are calculated using the interaction graph and P, and a new matrix, P’ is generated from E. If P’ is equal to P, convergence has been reached. If not, the process is repeated by updating P with a combination of P and P’. Once convergence is reached, the final energies matrix and probabilities matrix is used to generate the Boltzmann weights of each backbone position, which is then used to average all the backbone specificity profiles together. This specificity profile is divided by the background specificity profile to reach the final predicted specificity profile.

To predict the specificity profile, we consider an ensemble of peptide backbone conformations bound to a receptor. For each peptide backbone conformation, we simultaneously sample all rotameric conformations of all amino acids at all peptide residue positions while keeping the receptor backbone and sidechains in their crystallographic conformations. The sidechain conformations at a given peptide position are sampled in the “mean field” of all other sidechain conformations at all other positions and (fixed) receptor residues, as described in Methods. Next, the contribution of each peptide backbone conformation at each peptide position is accounted for by Boltzmann averaging the mean-field specificity profile solution obtained in the previous step. The final specificity profile is constructed by combining these individual predictions. While the sequence specificity prediction described here can be performed using any (pairwise decomposable) energy function, we implemented our prediction method in the context of the Rosetta modeling suite, thus combining its sophisticated energy function with the speed of mean-field sampling (Fig 1).

### Rationale for choice of benchmark datasets

To test our MFPred method, we sought to first recapitulate experimentally determined specificity profiles of a variety of PRDs. We chose PRDs where both structural as well as specificity information has been experimentally determined. We focused primarily on protease enzymes for methodology development, and tested the generality of our approach with previously developed benchmarks for multispecificity prediction on PRDs such as a kinase enzyme, and SH3, SH2, MHC, and PDZ domains.

#### Protease set

We benchmarked our method on four protease enzymes that had both high-resolution crystal structures with a bound peptide in the Protein Data Bank (PDB) and experimental cleavage data (see Methods for details). The chosen proteases represent the vast diversity seen in structural fold, biological function, and mechanism of action amongst the protease enzyme family (S2 Fig). Additionally, there is a mix of highly conserved and less specific positions among their specificity profiles, thus enabling us to determine how well MFPred performs with regard to varying degrees of flatness in the experimental specificity profile.

#### Testing on protein-recognition domains

To test the generality of the MFPred method, we curated a dataset consisting of a variety of non-protease PRDs that had high-resolution crystal structures as protein-peptide complexes in the PDB and experimental binding specificity data available. We tested fourteen PRDs that comprise five classes of PRDs: kinases, SH2 domains, SH3 domains, PDZ domains, and MHC-I proteins. Including these diverse domains allows us to test the method on a range of underlying recognition modes, binding affinities and specificities; while proteases bind with relatively high dissociation constants to their substrates (K_M_ ~10 uM), SH2 domains have been known to bind with dissociation constants as low as 0.3 nM [26].

The binding specificities and mechanisms for each of these domains are distinct, thereby adding to the diversity of the test set. PDZ domains bind up to 7 C-terminal residues in a highly specific manner [7]. SH3 domains bind proline-rich regions that often form PPII helices [10]. SH2 domains show a preference for pTyr-containing peptides [27], while the context surrounding the pTyr residue determines the specificity of the peptide towards a distinct SH2 domain [28]. Kinases are one of the largest families in the eukaryotic genome and share a common fold that allows for the binding of ATP and a Ser, Thr, or Tyr residue-containing substrate [29]. Finally, MHC-I domains bind short pathogenic peptides to be presented to cytotoxic T lymphocytes (CTLs). MHC-I domains are promiscuous and may bind many peptides; generally, one or two substrate positions are conserved, while others are tolerant to mutations [30].

### Choosing metrics for evaluation of prediction accuracy

We evaluated the performance of MFPred by quantifying the differences between predicted and experimentally determined specificity profiles using several metrics (see S1 Note for detailed descriptions of these metrics). Four of these metrics, the cosine similarity, Frobenius norm, average absolute distance (AAD) and Jensen-Shannon divergence (JSD) are correlated, as shown in S3 Fig. The Frobenius norm and AAD are distance-based metrics that have been used previously to compare profiles [21,22]. The Frobenius norm is more sensitive to flatness in the specificity profile than the AAD (S4 Fig). Additionally, we evaluated the profiles by their cosine similarity, which is another distance-based metric that is less sensitive to flatness than either AAD or Frobenius norm. It falls between 0 and 1, where 0 denotes a random prediction and 1 denotes a perfect prediction. The Jensen-Shannon divergence (JSD) has also been used in the past to evaluate profiles [21] and is less distance-based. We used cosine distance as the general score of a profile, as it is easy to visualize and interpret. It falls between 0 and 1, where 0 denotes a random prediction and 1 denotes a perfect prediction. For each position, we evaluated the significance of its JSD score by scoring 100,000 random profiles against the experimental profile and thus determining the *p*-value of the JSD score (see S1 Note for details).

We also used a second metric as a general score for each profile: area under the ROC (receiver operating characteristic) curve (AUC) is a non-distance-based metric that evaluates predictions based on their ranking more tolerated amino acids correctly [22]. It is relatively unaffected by flatness (S4 Fig) but will not evaluate well if either the experimental or predicted profile is close to uniform. It is not correlated with the above metrics. Additionally, we developed a new metric, Score Sequence AUC Loss (SSAL), which encapsulates the efficacy of the predicted specificity profile in differentiating between substrates which are recognized and cleaved by a given protease (cleaved sequences) and substrates which are not cleaved by that protease (uncleaved sequences). A perfect prediction scores an SSAL of zero. It does not correlate well with any other metric (S3 Fig).

### Recapitulation of protease specificity profiles

**Fig 2:**
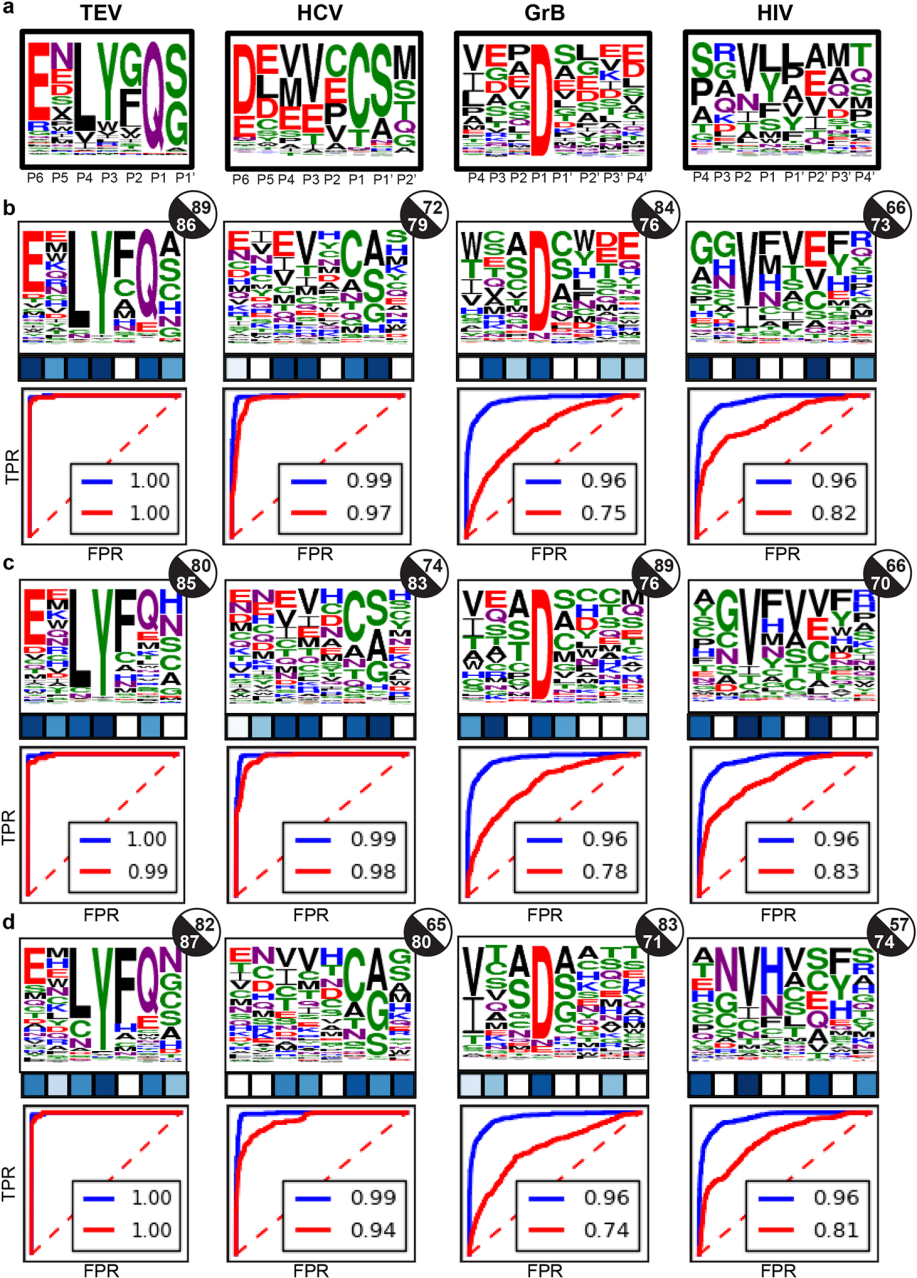
Comparison of backbone ensemble generation methods. **(a)** Experimental specificity profiles. **(b)** MFPred on FastRelax backbone ensemble. The *p*-value of the JSD for a given position is represented by the color of the square under that position; white denotes a *p*-value > 0.5 and dark blue denotes a *p*-value of 0. A given circle to the right of a profile represents the cosine similarity (white) and AUC (black) of that profile. The ROC plots beneath each profile depict the SSAL calculation *via* the experimental ROC (blue) and predicted ROC (red) with their respective AUC values. **(c)** MFPred on FlexPepDock backbone ensemble. **(d)** MFPred on Backrub backbone ensemble.

Proteolysis is a multi-step reaction which involves substrate peptide binding, the formation of a tetrahedral intermediate (acylation) and hydrolytic cleavage of the tetrahedral intermediate (deacylation). We have previously found that modeling a near-attack conformation for the acylation step was successful in discriminating between known cleaved and uncleaved peptides [31]. Therefore, starting from structures of protease-substrate complexes in a near-attack conformation, we performed MFPred-based specificity prediction. We found that MFPred robustly recapitulates protease specificity profiles (Fig 2B) in our benchmark set. The cosine similarities of the entire profiles range from 0.66 to 0.89, AUC ranges from 0.73 to 0.86, and SSAL ranges from 0.21 to 0.002. Out of 31 substrate positions across the protease dataset, 20 were predicted with a significant JSD *p*-value. The best prediction is obtained for the common biotechnologically used protease TEV-PR. The predicted profile has a high cosine similarity of 0.89 (1 would be a perfectly accurate prediction). The primarily steric and hydrogen-bonding-based nature of molecular recognition at TEV-PR-substrate interfaces is well suited to the strengths of the Rosetta energy function underlying MFPred. Similarly, the profiles of HCV protease and granzyme B (GrB) protease are also generally recapitulated with a high degree of accuracy, except for positions with no marked preference for specific amino acids (flat positions)–positions P5 and P2 in HCV protease and positions P4, P1’, and P2’ in granzyme B protease. We attribute the lack of correlation at these flat positions to small errors in energy evaluations being equivalent to the size of the energy gaps being modeled, thus leading to erroneous ranking. Challenges in measuring prediction accuracy at flat positions have indeed been noted before [22].

The worst performance among the proteases in the benchmark set is observed for the prediction of HIV protease-1 (HIVPR1) specificity. This protease is known to have a relaxed specificity profile, with preference for small hydrophobic residues at P1 and P1’ positions. The cavity of HIV protease-1 is large and peptides may adopt large variations in backbone conformation depending on their sidechains. Additionally, substrate binding involves flexibility on the protease side, with two loops (“flaps”) that are mobile and close over the binding pocket. Incorporation of greater backbone flexibility on both the receptor and peptide parts of the HIVPR1-peptide interface may help improve predictions, as previously observed by us and others [31–33].

### Modeling backbone flexibility is key for prediction accuracy

To determine the contribution of modeling backbone flexibility to the accuracy of prediction and to investigate if backbone sampling could be optimized for specificity prediction, we generated MFPred profiles with different levels of backbone flexibility.

First, we found that predictions generated by starting from a single crystallographically-determined backbone structure for the peptide led to poor accuracy for HCV and HIV proteases (panels f,h in S6 Fig), indicating that incorporating peptide backbone diversity is a key requirement for the observed accuracy of prediction. Second, we generated peptide backbone ensembles by threading on a varying number of known substrate (cleaved) peptides using three different Rosetta-based backbone sampling protocols (FastRelax [34], FlexPepDock [35], and Backrub [36]) separately to further diversify the peptide backbone ensemble. In each case, geometric constraints [31] were used to limit the scissile peptide bond to a near-attack conformation and the catalytic residues to an active conformation. The MFPred simulations were then performed on all backbone ensembles and their results were compared to each other (Fig 2).

While the algorithm is relatively robust to the method of backbone generation as long as scissile bond geometry is maintained, the FastRelax (FR) protocol has a small improvement in overall performance over the FlexPepDock (FPD) protocol, with 20 significant *p*-values (out of 31) for FR vs. 19 for FPD, and FPD has a minor increase in overall performance over Backrub (BR), with 19 significant *p*-values for FPD vs. 18 for BR. The profile for TEV-PR is predicted best by FR, due to better prediction of Q at P1 and S at P1’. In the case of HIV protease-1, FR recapitulates the profile better than FPD and BR do. However, the performance of FPD is marginally better than that of FR and significantly more accurate than that of BR in the cases of HCV protease and granzyme B protease.

To determine how MFPred accuracy depends on the number and sequences of known cleaved substrates used to generate the backbone ensemble, we generated a peptide backbone conformational ensemble that was independent of peptide sequence. For all positions on the peptide backbone, we enumerated every combination of phi/psi dihedral angles that were x-15, x, and x+15, where x is the dihedral angle of the relaxed crystal structure peptide backbone. The resulting structures were filtered to remove those with clashes and to preserve hydrogen-bond interactions. The remaining structures were further clustered by all-heavy-atom RMSD of the peptide residues (see S2 Note for details) and MFPred was performed on the cluster centers. The resulting predictions are significantly less accurate than those of FR, FPD, or BR (S5 Fig), indicating that successful prediction requires a backbone ensemble that is optimally positioned in the binding site for cleavage.

As a second test of the dependence of MFPred on the cleaved sequence information, we threaded five known uncleaved (*i*.*e*., not bound by the protease in a productive conformation) sequences on the peptide backbone and then performed FastRelax on the resulting structures. The prediction accuracy of MFPred decreased on these structures (S5 Fig), to the extent that the specificity profiles are almost uniform. Therefore, diversifying the peptide structure in suboptimal sequence space led to worse predictions than those obtained while diversifying it without any sequence information.

Next, to determine the impact of starting from bound complexes to generate MFPred predictions, we performed MFPred simulations on apo structures of two proteases: HCV NS3 protease and HIV protease-1 (S12 Fig). As HIV protease-1 has two flaps that can assume either a closed or open form [37], we used both a ‘closed apo’ structure and an ‘open apo’ structure for our simulations. In each case the protease all-atom RMSD between bound and open states, as determined by PyMol [38], were 1.04 Å, 1.85 Å, and 2.00 Å. In all three cases, MFPred accuracy was higher when starting from the bound complex compared to the apo state. While the number of significant *p*-values remains similar, the overall cosine similarities, AUC, and SSAL decreased for the apo structure-based simulations. Additionally, the information content decreased significantly for the apo structures of HIV (0.72–0.74 bits) as opposed to the bound complex (1.18 bits). Overall, the prediction accuracies between apo and bound states were more similar for the HCV protease where small backbone changes in the protease are incurred upon binding, compared to HIV protease where larger differences in prediction accuracy were apparent. These results suggest that especially in cases where there is significant backbone conformational change in the receptor upon peptide binding, such as the HIV protease, the incorporation of receptor flexibility may be needed for maintaining MFPred accuracy.

Finally, to investigate the dependence of performance accuracy on the number of known cleaved (recognized) sequences, we executed MFPred simulations on backbone ensembles generated from differing numbers of starting peptide sequences threaded on to the crystallographic backbone conformation. We varied the number of sequences used to generate the backbone ensemble from one sequence to five sequences to ten sequences to all known sequences in the benchmark set. We found that MFPred is highly dependent on *N*, the number of cleaved sequences used, when *N* is small (panels e-h in S6 Fig). However, as *N* increases, this effect is decreased. For TEV-PR and HCV protease, which have relatively few sequences (68 and 198 respectively), the prediction accuracy plateaus after ten sequences, although in some cases it may fluctuate slightly from five to ten to all sequences. However, for granzyme B and HIV proteases (356 and 374 cleaved sequences respectively), the accuracy of MFPred has a minor increase from ten to all sequences. Thus, there is a near-maximum of accuracy for each system; once that point of diminishing returns has been reached, incorporating more cleaved sequences does not lead to significant increases in the accuracy.

Besides determining that the level of backbone sampling was optimal for prediction, we also optimized sidechain sampling (S3 Table). Using an older version of the rotamer library (2002) [39] decreased scores for all systems. Increasing the fineness of rotamer chi-angle sampling or removing the starting sidechain conformation from the rotamer sampling had little impact on the results. Packing protease sidechains around the peptide (between distances of 4–8 Angstroms) decreased the accuracy of the results. This may be explained by the finding that hot spot residues at protein-protein interfaces often adopt strained rotamer configurations [40]; packing protease interface sidechains while designing peptide residues within MFPred may force protease sidechains to adopt conformations that are unfavorable for productive substrate binding.

### Comparison of MFPred with other structure-based approaches

**Fig 3:**
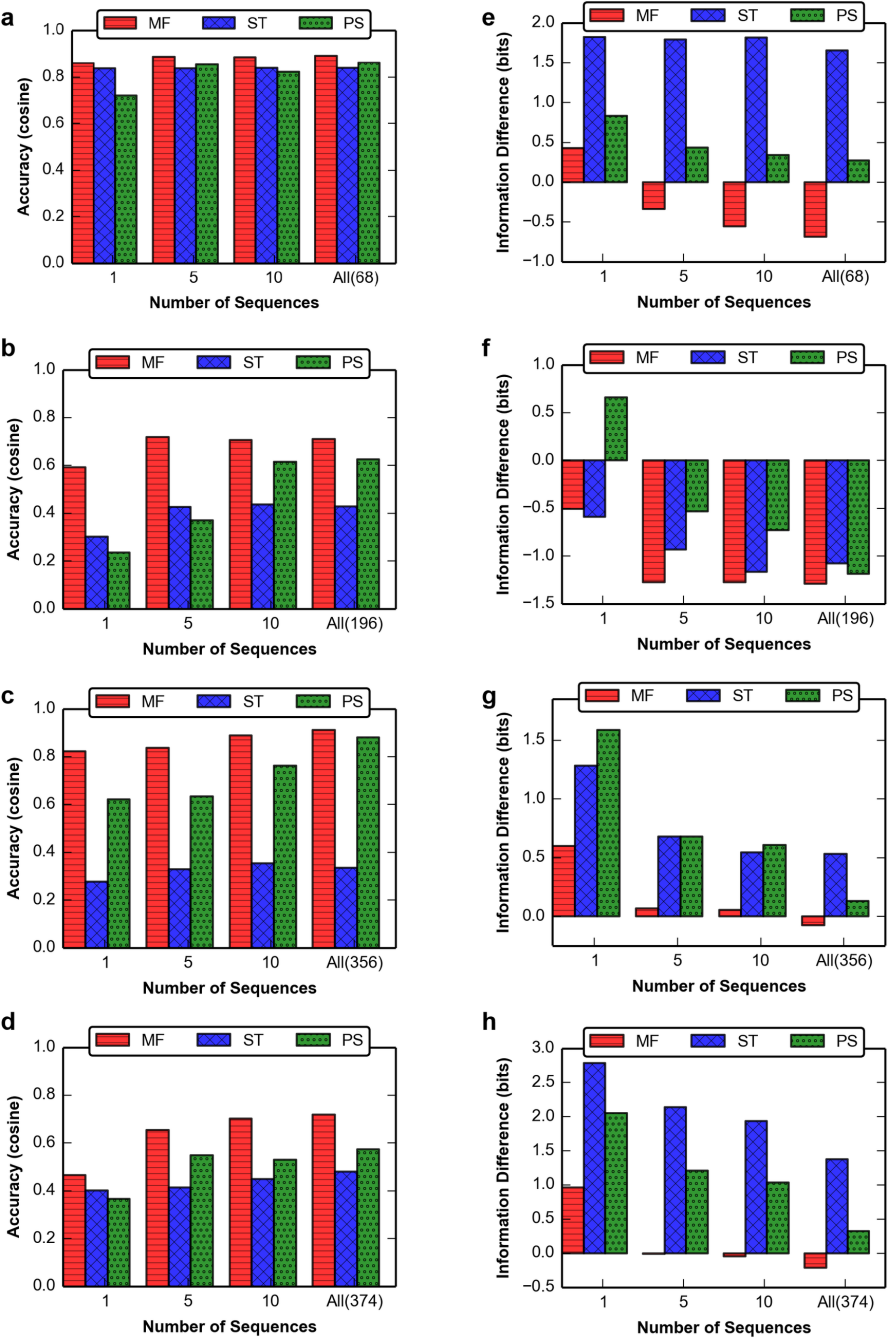
Number of sequences vs. accuracy and information for methods of profile prediction. **(a)-(d)** Number of sequences vs. accuracy for TEV, HCV, GrB, and HIV, respectively. Number of sequences is varied over 1-5-10-All experimentally derived sequences, which is different for each protease. **(e)-(h)** Number of sequences vs. information content (i.e. shape of profile) difference for TEV, HCV, GrB, and HIV, respectively. Information difference is equal to the predicted bits minus the experimental bits. An information difference that is close to zero approximates the experimental information content well; a highly positive information difference indicates a more peaked predicted than experimental profile while a highly negative information difference denotes a flatter predicted than experimental profile.

We compared our results to the two previously developed methods for specificity prediction that have been implemented in the Rosetta software. MFPred performed with comparable or greater accuracy than the sequence_tolerance [22] and pepspec [21] methods (Table 1). Additionally, MFPred was between 23-fold to 120-fold faster than the pepspec method and between 154-fold to 1154-fold faster than the sequence_tolerance method, depending on the number of peptide backbone conformations and rotamers (Table 1). For comparative benchmarking purposes, simulations were performed using a single AMD Opteron 6276 2.3 GHz processor. Furthermore, MFPred is more accurate on single backbones and smaller backbone ensembles than the other two methods; when performed on a backbone ensemble generated from five substrate sequences, MFPred predicts 19 out of 31 positions with a significant *p*-value, whereas only 11 of the positions predicted by sequence_tolerance and 8 of the positions predicted by pepspec yield significant *p*-values (S7 Fig). When executed on a single backbone conformation, MFPred predicts 12 positions with a significant *p*-value, while both sequence_tolerance and pepspec predict only 8 positions with a significant *p*-value. Both sequence_tolerance and pepspec are designed to be used with larger peptide ensembles–their success is dependent on a diverse backbone ensemble–and, as expected, their prediction accuracy increases as the number of backbones in the ensemble rises (Fig 3A–3D), with sequence_tolerance predicting 15 significant positions and pepspec predicting 16 significant positions on the backbone ensemble generated from all cleaved sequences (S8 Fig). When performed on this expanded backbone ensemble, MFPred prediction accuracy was also higher, with 25 significant predictions. Thus, compared to two state-of-the-art existing methods, MFPred-based predictions are of comparable or higher accuracy, and can be obtained with 10-1000-fold higher computational efficiency.

**Table 1:** Results of all methods—MFPred (MF), sequence_tolerance (ST), and pepspec (PS)—on variously-sized backbone ensembles.

Protease	Method	#Seq	Time(m)	Cosine	Frob	AAD	JSD	AUC	SSAL	Bits
**TEV**	**MF**	1	0.18	0.86	1.06	0.04	0.22	0.87	0.00	0.43
** **	** **	5	0.80	0.89	0.85	0.04	0.21	0.86	0.00	-0.34
** **	** **	10	2.08	0.88	0.86	0.04	0.20	0.91	0.00	-0.55
** **	** **	All (68)	11.97	0.89	0.84	0.03	0.20	0.91	0.00	-0.69
** **	**ST**	1	195.65	0.84	1.49	0.04	0.28	0.83	0.00	1.82
** **	** **	5	923.91	0.84	1.49	0.04	0.28	0.84	0.00	1.79
** **	** **	10	1827.32	0.84	1.49	0.04	0.28	0.85	0.00	1.82
** **	** **	All (68)	12333.94	0.84	1.44	0.04	0.28	0.84	0.00	1.65
** **	**PS**	1	17.46	0.72	1.50	0.05	0.36	0.81	0.01	0.83
** **	** **	5	96.01	0.85	1.06	0.04	0.24	0.92	0.00	0.44
** **	** **	10	189.43	0.82	1.17	0.04	0.24	0.85	0.00	0.34
** **	** **	All (68)	1290.41	0.86	1.04	0.03	0.21	0.86	0.00	0.27
**HCV**	**MF**	1	0.68	0.59	1.37	0.06	0.35	0.77	0.08	-0.51
** **	** **	5	3.61	0.72	1.13	0.05	0.31	0.79	0.02	-1.28
** **	** **	10	7.14	0.71	1.15	0.05	0.30	0.82	0.02	-1.28
** **	** **	All (196)	132.15	0.71	1.14	0.05	0.29	0.84	0.02	-1.29
** **	**ST**	1	115.04	0.30	1.77	0.07	0.53	0.63	0.30	-0.59
** **	** **	5	574.01	0.43	1.54	0.06	0.46	0.68	0.21	-0.93
** **	** **	10	1101.15	0.44	1.49	0.07	0.44	0.70	0.17	-1.16
** **	** **	All (196)	22239.05	0.43	1.51	0.07	0.44	0.67	0.17	-1.08
** **	**PS**	1	17.78	0.24	2.19	0.08	0.63	0.61	0.34	0.66
** **	** **	5	91.68	0.37	1.69	0.07	0.55	0.55	0.20	-0.53
** **	** **	10	171.30	0.61	1.30	0.06	0.39	0.73	0.05	-0.73
** **	** **	All (196)	3462.64	0.63	1.26	0.06	0.36	0.71	0.05	-1.19
**GrB**	**MF**	1	0.34	0.82	0.85	0.04	0.23	0.71	0.20	0.60
** **	** **	5	2.39	0.84	0.73	0.04	0.20	0.76	0.21	0.07
** **	** **	10	5.24	0.89	0.60	0.03	0.17	0.80	0.17	0.06
** **	** **	All (356)	145.63	0.91	0.53	0.03	0.13	0.87	0.15	-0.08
** **	**ST**	1	114.80	0.28	2.02	0.07	0.46	0.76	0.26	1.29
** **	** **	5	544.28	0.33	1.71	0.06	0.35	0.78	0.26	0.68
** **	** **	10	1109.45	0.35	1.62	0.05	0.31	0.82	0.17	0.55
** **	** **	All (356)	39036.17	0.34	1.67	0.05	0.32	0.84	0.21	0.53
** **	**PS**	1	19.58	0.62	1.45	0.06	0.51	0.61	0.38	1.59
** **	** **	5	101.24	0.63	1.15	0.06	0.39	0.70	0.34	0.68
** **	** **	10	203.69	0.76	0.99	0.05	0.29	0.78	0.27	0.61
** **	** **	All (356)	6814.15	0.88	0.64	0.03	0.17	0.86	0.18	0.13
**HIV**	**MF**	1	0.23	0.47	1.55	0.06	0.42	0.66	0.17	0.96
** **	** **	5	1.29	0.65	0.96	0.05	0.27	0.73	0.14	-0.01
** **	** **	10	3.15	0.70	0.88	0.04	0.23	0.78	0.08	-0.04
** **	** **	All (374)	110.65	0.72	0.82	0.04	0.21	0.81	0.05	-0.21
** **	**ST**	1	92.37	0.40	2.48	0.08	0.64	0.62	0.19	2.78
** **	** **	5	453.18	0.41	2.20	0.07	0.57	0.67	0.24	2.14
** **	** **	10	907.90	0.45	2.05	0.07	0.51	0.73	0.16	1.93
** **	** **	All (374)	34090.45	0.48	1.81	0.06	0.42	0.73	0.14	1.38
** **	**PS**	1	23.05	0.37	2.13	0.07	0.60	0.59	0.22	2.05
** **		5	109.77	0.55	1.54	0.06	0.40	0.69	0.11	1.21
** **		10	218.41	0.53	1.51	0.06	0.39	0.70	0.16	1.04
** **		All (374)	8134.56	0.57	1.23	0.05	0.28	0.76	0.10	0.33
Most Similar				1.00	0.00	0.00	0.00	1.00	0.00	0.00
Most Different				0.00	√(2n)^1^	0.06	1.00	0.00	1.00	4.32

^1^n refers to the number of positions in the profile

Besides informing us about the accuracy and speed of MFPred relative to existing methods, the comparison of MFPred to pepspec and sequence_tolerance allows us to categorize inaccuracies in MFPred predictions into those obtained from incorrect sequence sampling and those due to the Rosetta energy function or incomplete backbone conformational diversity. For example, MFPred on all cleaved backbones does not recover the experimentally determined high frequency for G at P2 of TEV-PR. Since both pepspec and sequence_tolerance also do not recover G at P2 with the same peptide backbone conformational ensemble, we attribute this inaccuracy to imperfections in the underlying Rosetta energy function and/or an incomplete peptide backbone ensemble used for prediction.

Generally, MFPred predicts lower information content (*i*.*e*. flatter shape) for the profiles than both sequence_tolerance and pepspec (Table 1, Fig 3E–3H). In the cases of granzyme B protease and HIVPR1, the predicted lower information content is reflective of the experimentally determined profiles; however, in the case of TEV-PR MFPred underestimates the information content relative to pepspec and sequence_tolerance. All protocols underestimate the information content of the profile of HCV protease. This underestimation may be due to an incomplete experimental dataset or sampling/scoring inaccuracies as discussed above. Overall, the difference between the predicted information content and the experimental information content was smaller for MFPred than for sequence_tolerance and pepspec, especially when performed with smaller backbone ensembles.

### Generalizing MFPred to other protein-recognition domains

**Fig 4:**
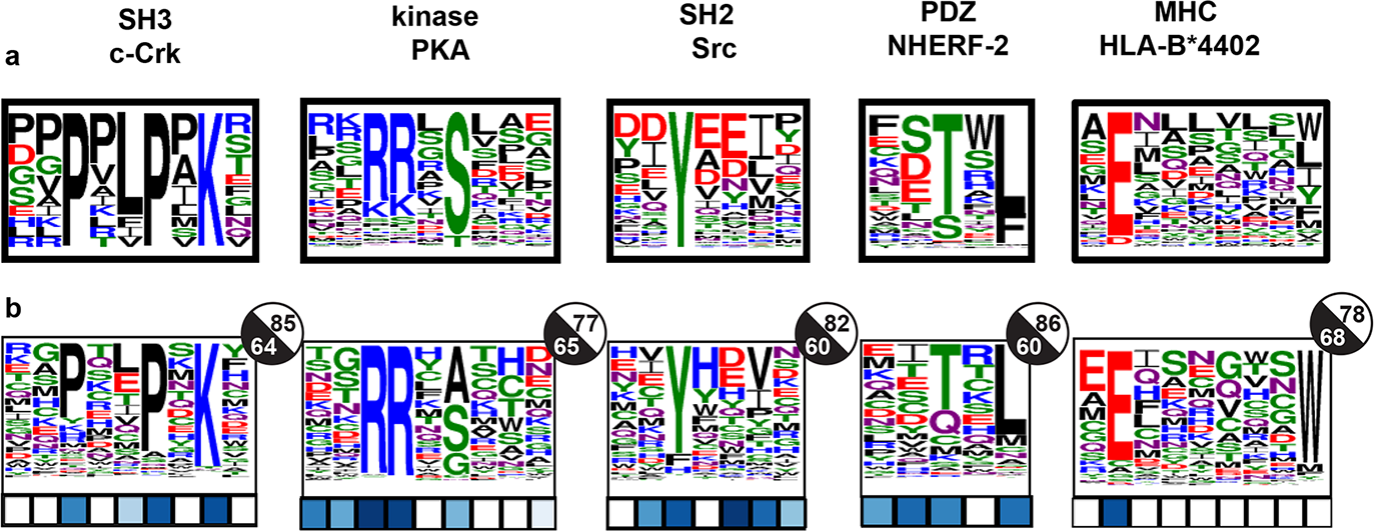
Generalize MFPred to PRD benchmark. **(a)** Experimental specificity profiles. **(b)** MFPred prediction. The *p*-value of the JSD for a given position is represented by the color of the square under that position; white denotes a *p*-value > 0.5 and dark blue denotes a *p*-value of 0. A given circle to the right of a profile represents the cosine similarity (white) and AUC (black) of that profile. For the PDZ domain, prediction was performed at a kT of 0.6, which was found to be optimal for PDZ domains.

To investigate the generality of our method for specificity prediction, we utilized the MFPred method to predict the specificity profiles for a variety of peptide-recognition domains: kinase, SH2, SH3, PDZ, and MHC domains. We achieved 17 significant *p*-values out of 31 positions and high cosine similarities (0.77–0.85) for three out of five PRD classes: PKA (kinase), Src (SH2), and c-Crk (SH3) domains (Fig 4). However, these three systems had lower AUCs (0.60–0.65). This may be due to the inadequacy of AUC as a metric for scoring positions that have low information content in the experimentally-derived profile; if few of the experimental amino acid frequencies are greater than 10%, the AUC reveals little about the prediction accuracy.

We predicted the specificity profiles of seven different PDZ domains: NHERF-2 PDZ2, PSD-95, AF-6 PDZ, Erbin PDZ, MPDZ-13, ZO-1 PDZ1, and DLG1-2 PDZ (Fig 4, S10 Fig). The specificity of NHERF-2 PDZ-2 was already predicted computationally by Zheng et al. [41], who were able to achieve good prediction via the use of CLASSY and FlexPepDock. King and Bradley previously predicted the specificity profile for PSD-95 computationally using pepspec [21], while the five other PDZ domain specificities were previously predicted by Smith and Kortemme via sequence_tolerance [22]. Six out of seven PDZ domains were predicted with medium to high accuracies, with cosine similarities of 0.63–0.86, AUCs of 0.60 to 0.88, and 25 out of 38 significant *p*-values. However, the prediction accuracy of the final PDZ domain, AF-6 PDZ was much lower, with a cosine similarity of 0.43, AUC of 0.59, and no significant *p*-values. This low accuracy may be due to the flexibility of the AF-6 PDZ domain, which has been known to bind in multiple binding modes and can be characterized as belonging to multiple classes of PDZ domain specificity [42,43]. Similar to the HIVPR1 case above, addition of receptor flexibility to MFPred may assist in AF-6 specificity profile recapitulation.

Finally, we tested the performance of MFPred on predicting MHC-I peptide recognition specificities. We selected four MHC-I domains with crystallographic structure availability and a large pool of known peptide binders [44]. The experimentally derived specificity profiles for the MHCs were highly conserved at one or two positions but relatively flat at others (Fig 4, S11 Fig). The MFPred predictions reflected this pattern: while 30 out of 36 positions had *p*-values that were not significant, due to the high tolerance of a diversity of amino acid at those positions, the cosine similarity of the predictions was high (0.63–0.78), reflecting good overall profile recapitulation (Fig 4, S11 Fig). These results indicate that robust and accurate predictions of the specificity profiles of a variety of peptide-recognition domains can be obtained using the MFPred approach, pointing to its wide applicability, especially for cases where receptor backbone flexibility is minimal. Improved modeling of backbone conformational diversity, an area where methodological improvements are needed [45], is likely to improve prediction accuracy further.

### Prediction of changes in multispecificity upon receptor mutation

**Fig 5:**
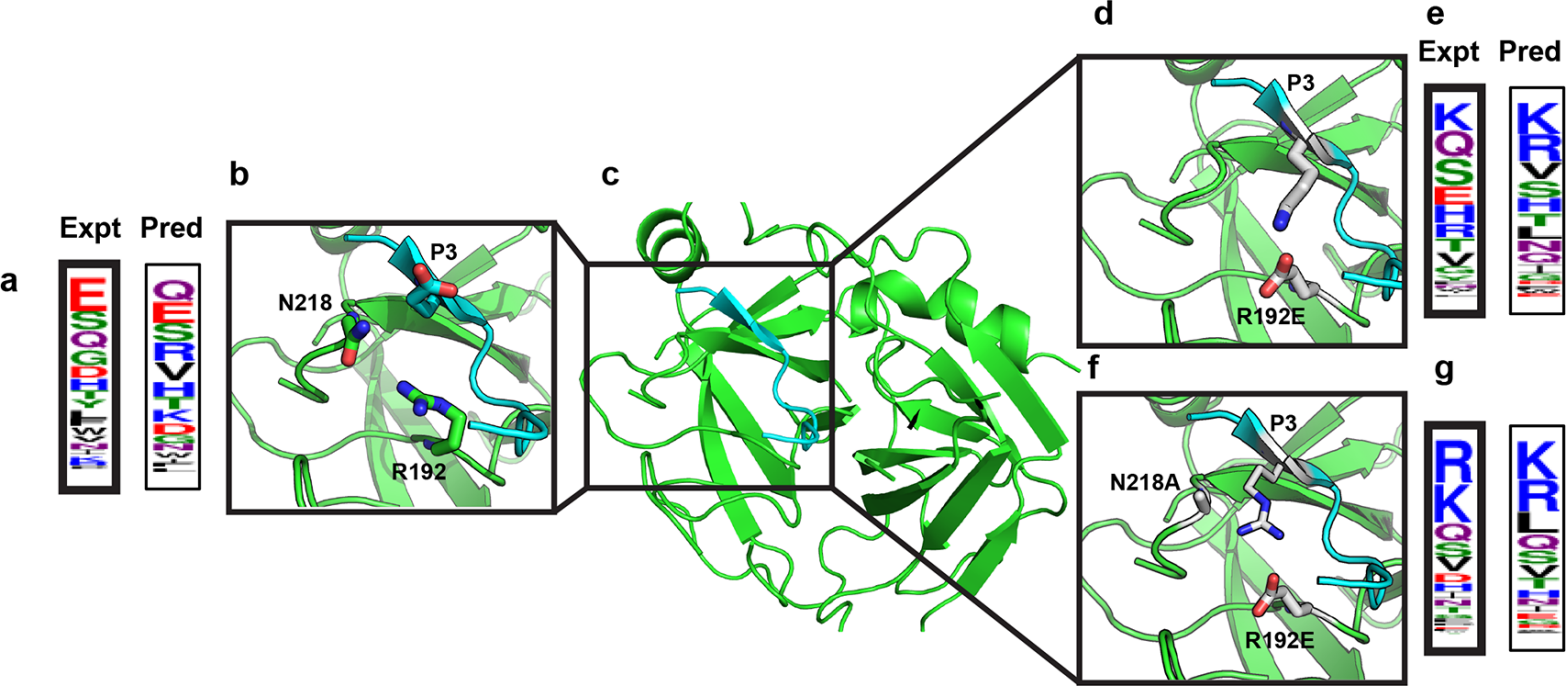
Proof-of-concept for design. **Changes in specificity profile upon granzyme B protease mutation are recapitulated by MFPred. (a)** Experimental (bold) specificity (average of Harris et al. [46] and Ruggles et al. [47]) and predicted P3 specificity for WT granzyme B protease. **(b)-(c)**, WT granzyme B protease structure. **(d)** R192E granzyme B protease active site. **(e)** Experimental specificity (bold) [46] and predicted P3 specificity for R192E granzyme B protease. **(f)** R192E/N218A granzyme B protease active site. **(g)** Experimental specificity (bold) [47] and predicted P3 specificity for R192E/N218A granzyme B protease.

When used to design receptors for and against specificity profiles, MFPred should be able to accurately recapitulate changes in specificity profiles due to protease mutations, when simulations are performed on a constant set of backbones. As a proof of concept, we predicted the changes in the specificity profiles of two variants of granzyme B protease for which altered multispecificity has been experimentally determined (Fig 5). R192E granzyme B protease and R192E/N218A granzyme B protease have been shown to have decreased specificity for glutamic acid and increased specificity for lysine and arginine at P3 [46,47]. To investigate whether MFPred can recapitulate mutant specificity profiles without changing the peptide backbone, we modeled the variants of granzyme B protease by performing the necessary mutations in Rosetta on the five FastRelaxed granzyme B protease backbones.

The MFPred-predicted specificity profile for the mutated structures accurately recapitulated the experimentally predicted specificity profile for the mutants. In the case of R192E, the change from a positively-charged arginine to a negatively-charged glutamic acid yields an increased frequency of positive amino acids such as lysine and arginine and a decreased frequency of negative amino acid glutamic acid. MFPred predicts the shift toward lysine and arginine and away from glutamic acid correctly, although it upweights the frequency of arginine and downweights the frequency of glutamic acid relative to the experimental profile. In the case of R192E/N218A, the shift towards arginine and lysine is even more pronounced in the experimentally-derived profile. Sterically, the mutation of N to A may allow for the longer sidechains of R and K (relative to E) to fit at P3. MFPred correctly predicts this shift as well. The sensitivity of MFPred to altered multispecificity at a given position due to a given receptor mutation should enable its use in designing for or against a given specificity profile.

## Discussion

Protein-peptide interactions underlie much of biology, and the ability to computationally manipulate these interactions would enable intervention in many biological processes. The rational design of receptor proteins, including enzymes that act upon peptide substrates, for and against peptide recognition specificity profiles is an open challenge. Such design would benefit from a specificity profile prediction technique that is both (i) rapid enough to be used in each step of the design process, and (ii) able to predict changed specificity for receptor variants with a constant peptide backbone conformational ensemble. The MFPred method developed here represents a step forward in achieving in both of these goals. MFPred is able to predict profiles for both proteases and a diverse set of PRDs, and it can recapitulate changes in the profile of variant granzyme B. This result sets the stage for application of the MFPred algorithm to enable the design of proteins for and against specificity profiles, by combining the MFPred algorithm with multi-state design [48].

The MFPred method, implemented in the context of the Rosetta software, performs specificity profile prediction with equivalent or better accuracy when compared to two previously developed methods (pepspec, sequence_tolerance) in the Rosetta framework, but with a significant decrease in run time (~10- to 1000-fold). Practically, this means that given a receptor variant and a peptide backbone ensemble, a specificity profile can be obtained, on a standard single processor, on a time-scale of seconds *vs*. hours required for other approaches. While pepspec and sequence_tolerance are less accurate on a smaller peptide backbone ensemble, MFPred is relatively robust to the size of the backbone ensemble. Additionally, MFPred can predict information content (determined from the amino acid frequency distribution at a given peptide position) better than other methods (Fig 3E–3H). The ability to recapitulate information content should enable design for a narrow or wide range of amino acid types at a given peptide position, thereby allowing greater control over binding selectivity. The speed, prediction accuracy on a small backbone ensemble, and robust recapitulation of information content of MFPred are due to the mean-field approach of MFPred: rather than attempt to enumerate many sequences on varying backbones, MFPred predicts a specificity profile by treating amino acid energies as a Boltzmann probability distribution. However, optimal sampling of the peptide backbone conformational space by MFPred does require some prior knowledge in the form of several (~5) recognized substrates, which is not required for pepspec or sequence_tolerance.

While MFPred can rapidly and consistently generate recognition profiles with high accuracy compared to experimental data, it was not possible to achieve a perfect prediction using MFPred. Several reasons may underlie these limitations of MFPred. First, our experimental dataset may be incomplete: it comprises various *in vitro* and *in vivo* sources in the literature, each of which may have their biases. *In vitro* experimental profiles vary with the definition of a cleaved sequence; when few sequences are included in this definition, the profile will converge on a few optimal sequences. *In vivo* experimental profiles are subject to biases due to biological factors [21]. Second, any specificity prediction challenge is composed of several, smaller problems–sampling the vast sequence space, sampling the significantly larger conformational space, and scoring the structures–each of contributes multiplicatively to the error-rate. In our study, the sequence sampling problem is solved by MFPred itself. As it is an approximation, MFPred may not sample the sequence space effectively; the free parameters, which are optimized for overall success, are sub-optimal for each system. This is especially true in the case of the temperature parameter, which we found to be the most system-dependent. Thus, application of MFPred to domain families that are not included in our benchmark set may require further system-specific optimization of model parameters to achieve comparable accuracy. In terms of structure sampling, our method of utilizing a small number of known recognized peptides to generate a backbone ensemble is an attempt to more efficiently sample the large backbone conformational space (which also determines sidechain sampling due to the use of a backbone-dependent rotamer library [49]); however, this space is so large, especially in the case of a flexible binding pocket such as the HIV protease-1, that sampling efficiency is still limited. The sampling of receptor backbone flexibility is also required in such cases, as evidenced by a decreased prediction accuracy when the apo-structure of the complex is used (S12 Fig). Finally, we score the structures using an empirical energy function (from Rosetta); subtle errors in the energy function may also contribute to the observed inaccuracies. As both conformational and sequence sampling in the MFPred approach rely on, and are limited by, the underlying rotamer library and energy function as implemented in Rosetta, improvements in these features [49,50] should yield higher accuracy predictions.

## Methods

### Inputs

#### Structure preparation

Crystal structures of the four protease-peptide complexes, fourteen protein-recognition domains, and three protease apo structures were procured from the Protein Data Bank (PDB) (S1 Table) [27,37,42,51–65]. Structures were filtered for a resolution equal to or lower than 2.8 Å and a bound peptide or peptidomimetic inhibitor. Active site mutations were reverted to the wild-type residues.

The selected crystal structures were optimized using Rosetta FastRelax to find a low energy structure, which was used as a starting point in further calculations. In the case of the protease enzymes, constraints were applied to catalytic residues during FastRelax to maintain active site geometry and keep the protease in a pre-transition-state near-attack conformation, and coordinate constraints were applied to the backbone to ensure that the enzyme did not unfold; we did not apply constraints in the general PRD benchmark, as constraints were found to decrease prediction accuracy in those cases. Peptide side chains and backbone were allowed to sample all degrees of freedom including rotation, translation, and rigid body orientation with respect to the protease. The models were scored with Rosetta’s talaris2013 energy function.

The apo crystal structures were aligned with the relaxed models of the protease-peptide complexes using PyMol [38], and the peptides from the protease-peptide complexes were placed within the apo models. The crystal structures were further optimized using Rosetta FastRelax as described above.

#### Experimental sequence profiles and cleaved/uncleaved sequences

The sequences of cleaved and uncleaved substrate peptides for each protease and bound peptides for each PRD were obtained as described in Table 2. For further details on the curation of the protease datasets, please see our recent study [31]. To generate a specificity profile for each protease, we first removed duplicates from the set of cleaved peptides and then calculated the frequency of each amino acid at each position. We followed the same procedure for the PRDs; however, we did not remove duplicates from those sets. The sequence sets are provided in S1 Dataset.

**Table 2:** Substrates for proteases and PRDs.

**Protease**	**# Cleaved**	**# Uncleaved**	**References**
TEV-PR	68	1520	• Kostallas et al. [66] • Boulware et al. [67]
HCV protease	196	1943	• Shiryaev et al. [68] • Rögnvaldsson et al. [69]
Granzyme B protease	353	1973	• Barkan et al. [70]
HIV-PR	374	1251	• Rögnvaldsson et al. [69]
**PRD**	**#Bound *in vitro***	**#Bound *in vivo***	**References**
c-Crk SH3-N	13	N/A	• Sparks et al. [10]
cAMP-dependent PKA	346	19	• PhosphoELM [71] • Schutkowski et al. [5]
Src SH2	13	117	• PepCyber [72] • Khati et al. [6]
PSD-95 PDZ3	93	2	• PDZBase [73] • Tonikian et al. [7]
NHERF-2 PDZ2	132	N/A	• Vouilleme et al. [8] • Stiffler et al. [9] • Tonikian et al. [7]
AF-6 PDZ	176	N/A	• Tonikian et al. [7]
Erbin PDZ	86	N/A	• Tonikian et al. [7]
MPDZ-13 (PDZ)	91	N/A	• Tonikian et al. [7]
ZO-1 PDZ1	71	N/A	• Tonikian et al. [7]
DLG1-2 (PDZ)	58	N/A	• Tonikian et al. [7]
HLA-A*0201 (MHC)	3273	N/A	• Vita et al. [44]
HLA-B*1501 (MHC)	1187	N/A	• Vita et al. [44]
HLA-B*4402 (MHC)	236	N/A	• Vita et al. [44]
HLA-B*4403 (MHC)	207	N/A	• Vita et al. [44]

### Backbone ensemble generation

We generated a flexible backbone ensemble by constructing models of the proteins bound to several cleaved sequences, and then diversifying those models via FastRelax [34], FlexPepDock [35], or Backrub [36] backbone sampling protocols, as described in detail below. For each protein, *N* cleaved sequences were chosen from the dataset by sorting the sequences in alphabetical order and then choosing evenly spaced sequences from the sorted dataset. Two alternative methods of picking cleaved sequences—randomly, or at even intervals from a set sorted by hamming distance from an arbitrarily chosen cleaved sequence—did not impact the results.

Then those *N* cleaved sequences were threaded onto the original FastRelaxed protein-peptide complex to create *N* structure-sequence models. Each model was subjected to 10 trajectories of FastRelax simulations, 10 trajectories of FlexPepdock refine simulations, or 10 trajectories of Backrub simulations, and the resulting 10 models were considered to be the backbone conformational ensemble. As we found that the FastRelax protocol was more accurate than FlexPepDock and Backrub, we used FastRelax alone in the final version of the protocol. The model was constrained to active catalytic geometry for the proteases; we did not apply constraints to the PRD systems. Finally, the *x* lowest-scoring models for each sequence (with *x* dependent on the protocol in question, and generally set as 1) were chosen as the final backbone ensemble.

### Mean-field algorithm

Various self-consistent mean-field theory-based methods have been developed for use in protein sidechain packing and design [74–81]. In the canonical self-consistent mean field theory-based method for protein sidechain packing as proposed by Koehl and Delarue [74], the energy landscape is investigated by using an effective energy potential to approximate the effects of all possible rotamers at all positions to be modeled. Thus, the mean-field energy of rotamer *r* occurring at position *i* is determined by Eq 1:

$$E(i,r)=e(i_{r})+\sum_{j=1,j\neq i}^{N}\sum_{s=1}^{K_{j}}e(i_{r},j_{s})P(j,s)$$ (1)

*e*(*i*_*r*_) represents the one-body energy of the rotamer, or the energy between a residue and the fixed components of the protein. *e*(*i*_*r*_,*j*_*s*_) represents the two-body energy between a rotamer *r* at position *i* and a rotamer *s* at position *j*. Energies are truncated at a threshold that we optimized as a free parameter. *P(j*, *s)* represents the probability of rotamer *s* occurring at position *j* and is initially given as 1/*K*_j_, where *K*_j_ is the total number of available rotamers at position *j* (obtained from a rotamer library).

A probability matrix (**P**) of size *N* × *K*_max_, where *N* is the number of positions to be analyzed and *K*_max_ is the maximum number of rotamers at any position, is used to model the probabilities of each rotamer occurring. Once the effective energy of each rotamer is determined using (1), the probability of each rotamer is:

$$P(j,s)=\frac{e^{-\beta E(j,s)}}{\sum_{x=1}^{K_{j}}e^{-\beta E(j,x)}}$$ (2)

*β* (= 1/*k*T) is also optimized as a free parameter. The algorithm iterates between the two equations until convergence is reached. We use a pre-calculated interaction graph in Rosetta [82] to store the one-body and two-body energies, which do not change between iterations, so the iteration is rapid. Convergence is improved with the use of a memory in the updating of **P**, so that the probability matrix after iteration *x* is given by *P*_*x*_ = λ*P*_*x*−1_ + (1−λ)*P*_*x*_, where λ is a free parameter between 0 and 1. Once convergence is reached, the probability matrix **P** can be used to obtain the probability for every rotamer.

We extended the algorithm for use with a flexible backbone and with any given amino acid alphabet. Given an ensemble of backbone conformations, the probability matrix **P** is calculated for each backbone using the canonical self-consistent mean field method, while allowing each position to take on any amino acid, so that the vector for that position contains all the rotamers for all amino acids at that position. P_*aa*_(*bb*, *i*), the probability of amino acid *aa* occurring at position *i* in backbone *bb*, is determined for all amino acids at all positions in all backbones:

Paa(bb,i)=∑r=1KaaPbb(i,r)Kaaγ∑x=120∑r=1KxPbb(i,r)Kxγ (3)

where *K*_aa_ is the number of rotamers available to amino acid aa at position *i*, and γ is a free parameter optimized to 0.8 in our implementation. Dividing the sum of probabilities over all rotamers for amino acid *aa* by $K_{aa}^{\gamma }$ thus corrects for cases where numerous rotamers of an amino acid artificially inflate the probability of a specific amino acid occurring (S1 Fig).The probability matrices for all backbones are then averaged together using a Boltzmann-weighting scheme in a two-step process. First, *E*_*bb*_(*i*,*aa*), the weighted sum of the energies for rotamers of amino acid *aa* at position *i* in backbone *bb*, divided by $K_{aa}^{\gamma }$, is calculated (Eq 4). Then *E*_*bb*_(*i*,*aa*) is used to find *W*(*i*), the probability of backbone bb occurring at position i (Eq 5). *M* is the number of (peptide) backbones in the ensemble.

$$E_{bb}(i,aa)=\frac{\sum_{r=1}^{K_{aa}}E_{bb}(i,r)P_{bb}(i,r)}{K_{aa}^{\gamma }}$$ (4)

$$W(i)=\frac{e^{-\beta \sum_{aa=1}^{20}E_{bb}(i,aa)}}{\sum_{s=1}^{M}e^{-\beta \sum_{aa=1}^{20}E_{s}(i,aa)}}$$ (5)

Finally, a weighted average P is determined and taken to be the predicted specificity profile for that protease:

$$P(i,aa)=\sum_{bb=1}^{M}P_{aa}(bb,i)W(i)$$ (6)

Thus, MFPred can be used for prediction of multispecificity for both one backbone and multiple backbone conformations.

### Parameter optimization of MFPred

To optimize four free parameters for MFPred (λ, γ, threshold, and *k*T), we enumerated all combinations of λ (0.25, 0.5, 0.75), γ (0, 0.2, 0.4, 0.6, 0.8, 1.0), threshold (5, 10, 50, 100, 250, 500), and *k*T (0.2, 0.4, 0.6, 0.8, 1.0). We selected 68 structures from the peptiDB (a peptide-protein complex database) [83] that met our criteria of having at least eight peptide residues. The structures were input into MFPred as a backbone ensemble and all combinations of the above parameters were tested. The resulting background specificity profiles were compared to the background residue distribution in the Rosetta database (S1 Fig, S9 Fig) and the combination of parameters with the lowest cosine distance from the known background distribution was chosen as our final set of parameters. While varying λ had little impact on the results, all other parameters had a significant, system-dependent impact on the results.

### Enrichment over background

Since the MFPred predictions include noise arising from limited sampling and the scoring function used (as mentioned above), we divided its predictions by the background profile to find the final prediction. The background profile was determined by averaging the frequencies of each position in the peptiDB profile. We divided each amino acid frequency in the initial predicted profile by the frequency of that amino acid in the background profile to find the final profile (S9 Fig).

### Software availability

MFPred is available as a RosettaScripts Mover within the master branch of Rosetta. Sample cases for how to use MFPred can be found in S2 Note and in online Rosetta documentation.

## Supporting information

S1 Fig The need for γ in the mean-field algorithm when averaging rotamers of an amino acid to find the probability of that amino acid. (a) Background amino acid composition as defined in Rosetta database (P_AA). This is the gold-standard which we attempted to match in our background profile generation (see Methods). (b) MFPred’s background prediction with γ = 0, i.e. the rotamer probabilities are simply summed to find the amino acid probability. Serine and threonine are overrepresented as the Rosetta Dunbrack library contains many more rotamers for S and T, and glycine and alanine are underrepresented due to having only one rotamer each. (c) MFPred’s background prediction with γ = 0.8 (current settings). This is closest to the P_AA distribution (Frobenius distance of 0.24). (d) MFPred’s background prediction with γ = 1.0, *i*.*e* the amino acid probability is simply the average of the rotamer probabilities. While this is better than γ = 0, alanine and glycine are now overrepresented and serine and threonine are underrepresented. Frobenius distance is 0.39.
(PNG)

S2 Fig Protease benchmark specificity profiles, models, active centers, and recognition modes. (a) Tobacco etch virus (TEV) protease is a cysteine protease displaying extensive hydrogen bonding at the protease-substrate interface. (b) Hepatitis C virus (HCV) NS3 protease, a serine protease, recognizes substrates via interfacial hydrogen bonding. (c) Granzyme B, a serine protease, recognizes substrates through electrostatic interactions. (d) Human immunodeficiency virus (HIV) protease I, a symmetric aspartyl protease, has been proposed to recognize substrates via the substrate-envelope hypothesis.
(PNG)

S3 Fig Specificity profile metric correlation. Correlation coefficients between pairs of metrics are shown in the upper diagonal while scatterplots are shown in the lower diagonal. Cosine similarities and AUC values are shown as 1 –cosine and 1 –AUC, respectively, so that a lower value represents a better prediction. Scatterplot points are colored by the number of bits in the predicted profile, with darker blue representing fewer bits, or more peaked profiles.
(PNG)

S4 Fig Profile shape affects evaluation metrics differently. (a) “Experimental” profile to compare to. (b) Each metric is affected differently by the shape of the profile (x-axis). Accuracy is normalized for all metrics so that the worst metric corresponds to one. Both AUC and cosine are subtracted from 1, as well. Cosine similarity varies slightly with regard to flatness of the profile, whether or not the most frequent amino acid is correct. Frobenius distance varies more than the cosine similarity; it decreases somewhat consistently with the shape of the profile. While AAD does not vary much with regard to flatness when the most frequent amino acid is incorrect, it decreases very quickly when the most frequent amino acid is correct. JSD also varies more when the most frequent amino acid is correct, although to a lesser extent than AAD. AUC is relatively unaffected by flatness; if the most frequent amino acid is incorrect, it is ~0.5 (or random), and if the most frequent amino acid is correct, it is zero.
(PNG)

S5 Fig Incorporating cleaved sequences into backbone ensemble generation improves MFPred’s accuracy. (a) Experimental specificity profiles. (b) Results of running MFPred on backbone ensemble of five cleaved sequences FastRelaxed. (c) Results of running MFPred on backbone ensemble generated by enumerating combinations of phi/psi angles (see paper for details). (d) Results of running MFPred on backbone ensemble of five uncleaved sequences FastRelaxed.
(PNG)

S6 Fig Number of sequences vs. accuracy and number of backbones vs. accuracy for methods of backbone ensemble generation. (a)-(d) Number of backbones per sequence vs. accuracy for TEV, HCV, Granzyme B, and HIV, respectively. Each protocol begins with five sequences, which are then relaxed using FR, FPD, or BR 1, 2, 5, or 10 times each. (e)-(h), Number of sequences vs. accuracy for TEV, HCV, Granzyme B, and HIV, respectively. Number of sequences is varied over 1-5-10-All experimentally derived sequences, which is different for each protease.
(PNG)

S7 Fig MFPred vs. other Rosetta prediction techniques on ensemble of five sequences. (a) Experimental specificity profiles. (b) MFPred. (c) pepspec. (d) sequence_tolerance.
(PNG)

S8 Fig MFPred vs. other Rosetta prediction techniques on ensemble of all sequences. (a) Experimental specificity profiles. (b) MFPred. (c) pepspec. (d) sequence_tolerance.
(PNG)

S9 Fig Enriching specificity profiles over background specificity profile improves accuracy. (a) Experimental specificity profiles. (b) Initial MFPred-predicted specificity profiles. (c) Specificity profiles divided by background specificity profile. (d) Background specificity profile.
(PNG)

S10 Fig MFPred prediction for six PDZ domains. (a,c) Experimental specificity profiles. (b,d) MFPred prediction. Prediction was performed at a *k*T of 0.6, which was found to be optimal for PDZ domains.
(PNG)

S11 Fig MFPred prediction for three MHC-I domains. (a) Experimental specificity profiles. (b) MFPred prediction.
(PNG)

S12 Fig Using structures of receptor-peptide complexes vs. apo structures improves the accuracy of MFPred. (a) Experimental specificity profiles. (b) MFPred prediction on receptor-peptide complexes. (c) MFPred prediction on HCV NS3 protease apo structure. (d) MFPred prediction on HIV protease 1 closed form apo structure. (e) MFPred prediction on HIV protease 1 open form apo structure.
(PNG)

S1 Table Details of model generation for four proteases and fourteen PRDs. (DOCX)

S2 Table Results of MFPred on different backbone ensembles. (DOCX)

S3 Table Effect of various Rosetta settings on MFPred predictions on five sequence backbones. (DOCX)

S1 Note Explanation of metrics. (DOCX)

S2 Note Supplementary software. (DOCX)

S1 Dataset Lists of cleaved/uncleaved/bound sequences. (XLSX)
