# Supplementary figures and images for: MFPred: Rapid and accurate prediction of protein-peptide recognition multispecificity using self-consistent mean field theory
Source: PLoS Comput Biol. 2017 Jun 26;13(6):e1005614. doi: 10.1371/journal.pcbi.1005614 (PMC5507473; doi:10.1371/journal.pcbi.1005614)

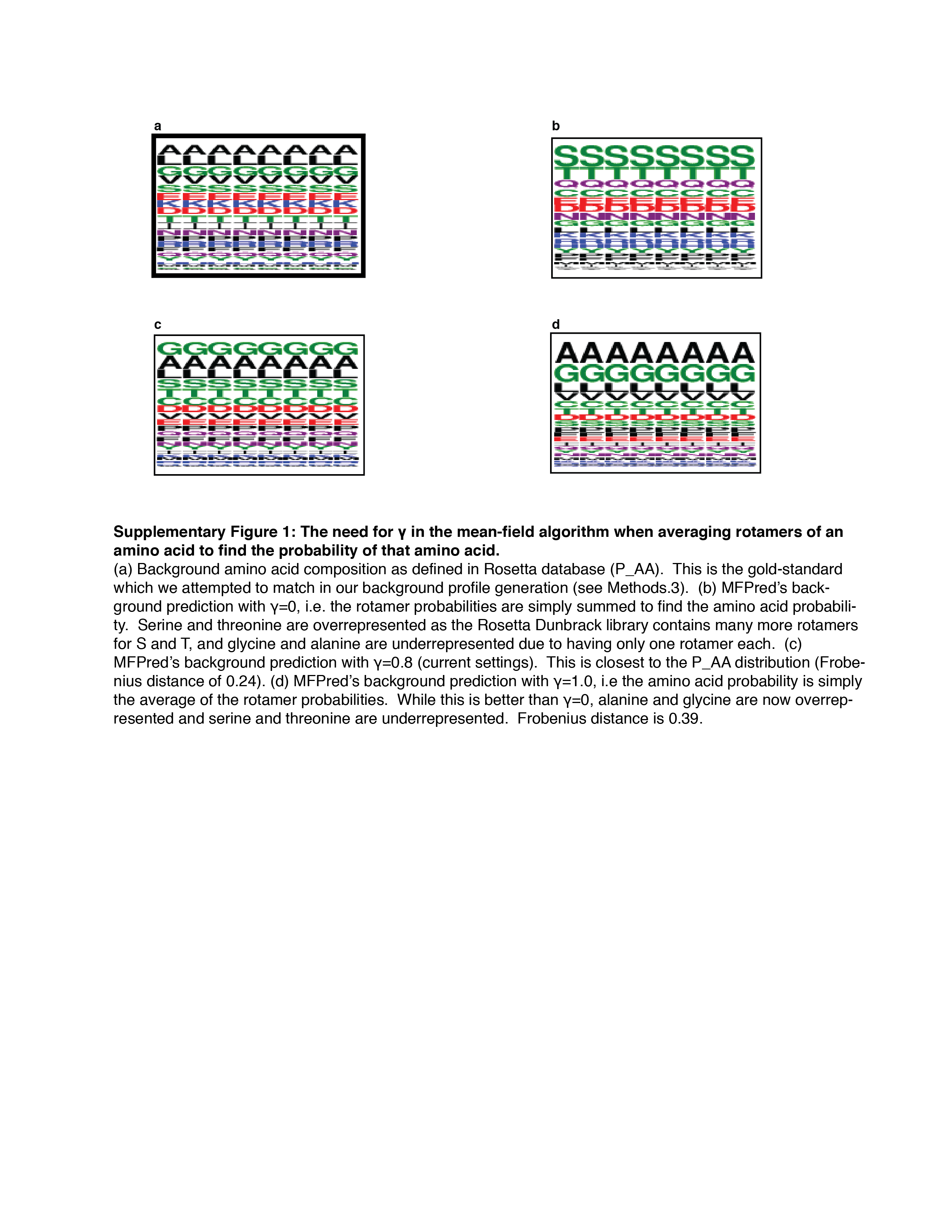

Supplement: S1 Fig — (a) Background amino acid composition as defined in Rosetta database (P_AA). This is the gold-standard which we attempted to match in our background profile generation (see Methods). (b) MFPred’s background prediction with γ = 0, i.e. the rotamer probabilities are simply summed to find the amino acid probability. Serine and threonine are overrepresented as the Rosetta Dunbrack library contains many more rotamers for S and T, and glycine and alanine are underrepresented due to having only one rotamer each. (c) MFPred’s background prediction with γ = 0.8 (current settings). This is closest to the P_AA distribution (Frobenius distance of 0.24). (d) MFPred’s background prediction with γ = 1.0, i.e the amino acid probability is simply the average of the rotamer probabilities. While this is better than γ = 0, alanine and glycine are now overrepresented and serine and threonine are underrepresented. Frobenius distance is 0.39. (PNG) [file pcbi.1005614.s001.png]

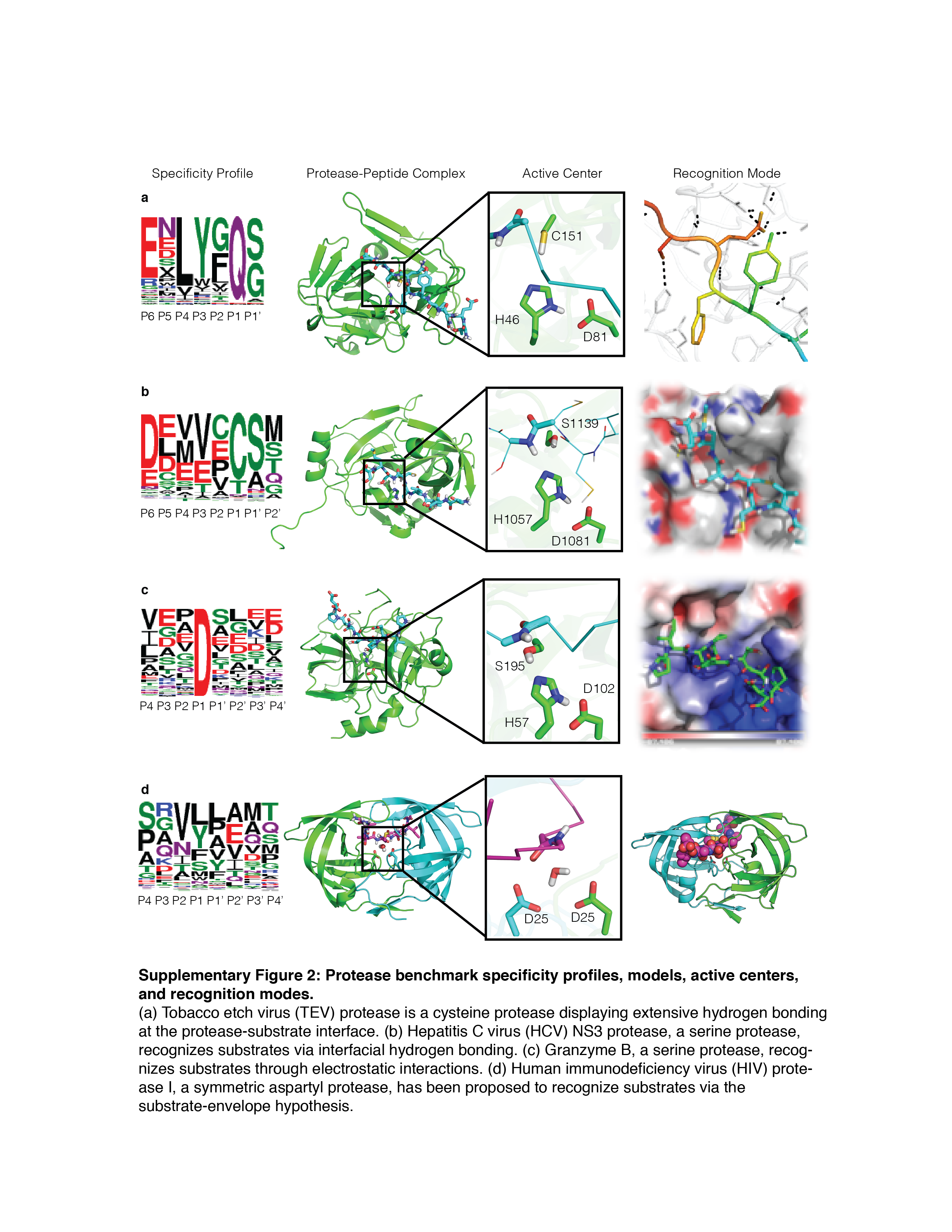

Supplement: S2 Fig — (a) Tobacco etch virus (TEV) protease is a cysteine protease displaying extensive hydrogen bonding at the protease-substrate interface. (b) Hepatitis C virus (HCV) NS3 protease, a serine protease, recognizes substrates via interfacial hydrogen bonding. (c) Granzyme B, a serine protease, recognizes substrates through electrostatic interactions. (d) Human immunodeficiency virus (HIV) protease I, a symmetric aspartyl protease, has been proposed to recognize substrates via the substrate-envelope hypothesis. (PNG) [file pcbi.1005614.s002.png]

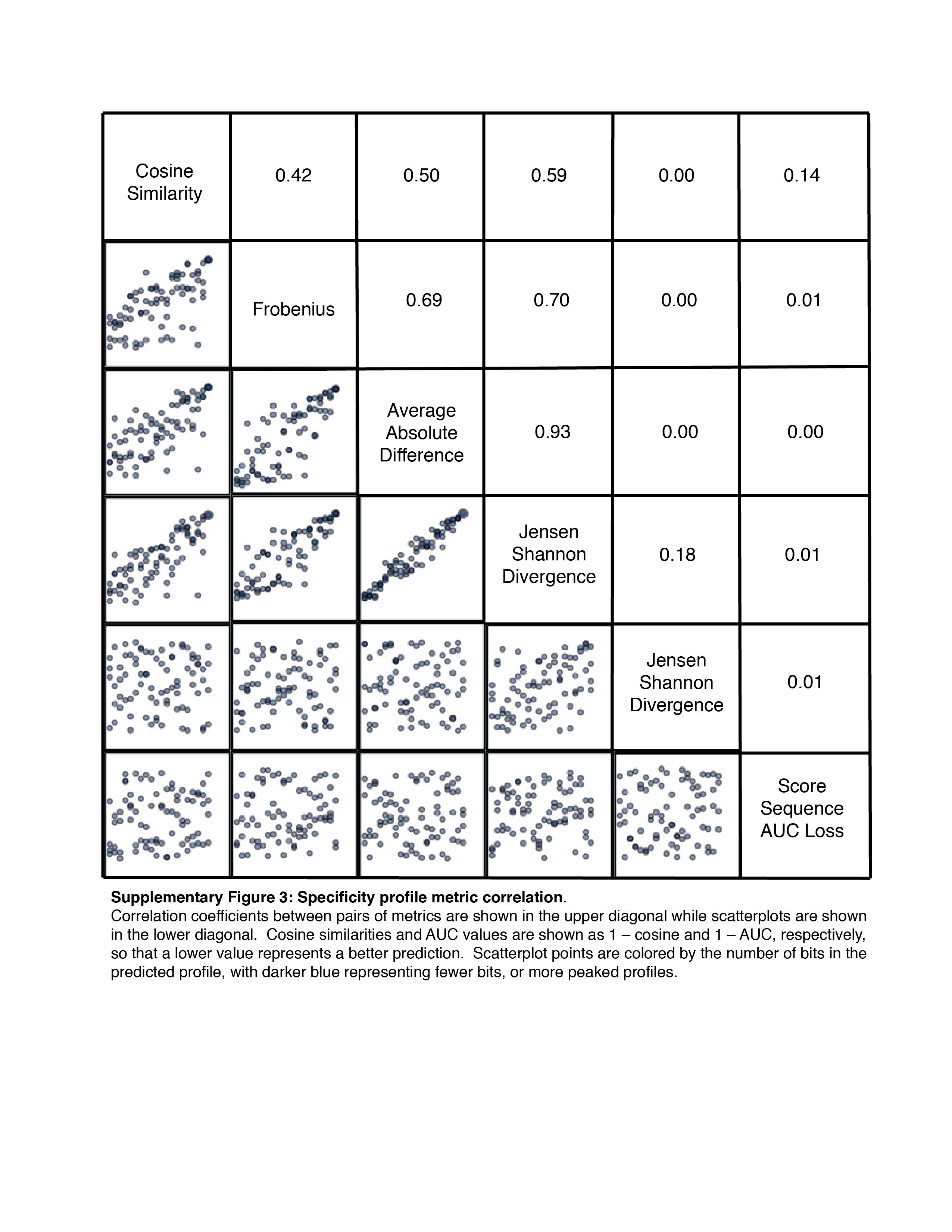

Supplement: S3 Fig — Correlation coefficients between pairs of metrics are shown in the upper diagonal while scatterplots are shown in the lower diagonal. Cosine similarities and AUC values are shown as 1 –cosine and 1 –AUC, respectively, so that a lower value represents a better prediction. Scatterplot points are colored by the number of bits in the predicted profile, with darker blue representing fewer bits, or more peaked profiles. (PNG) [file pcbi.1005614.s003.png]

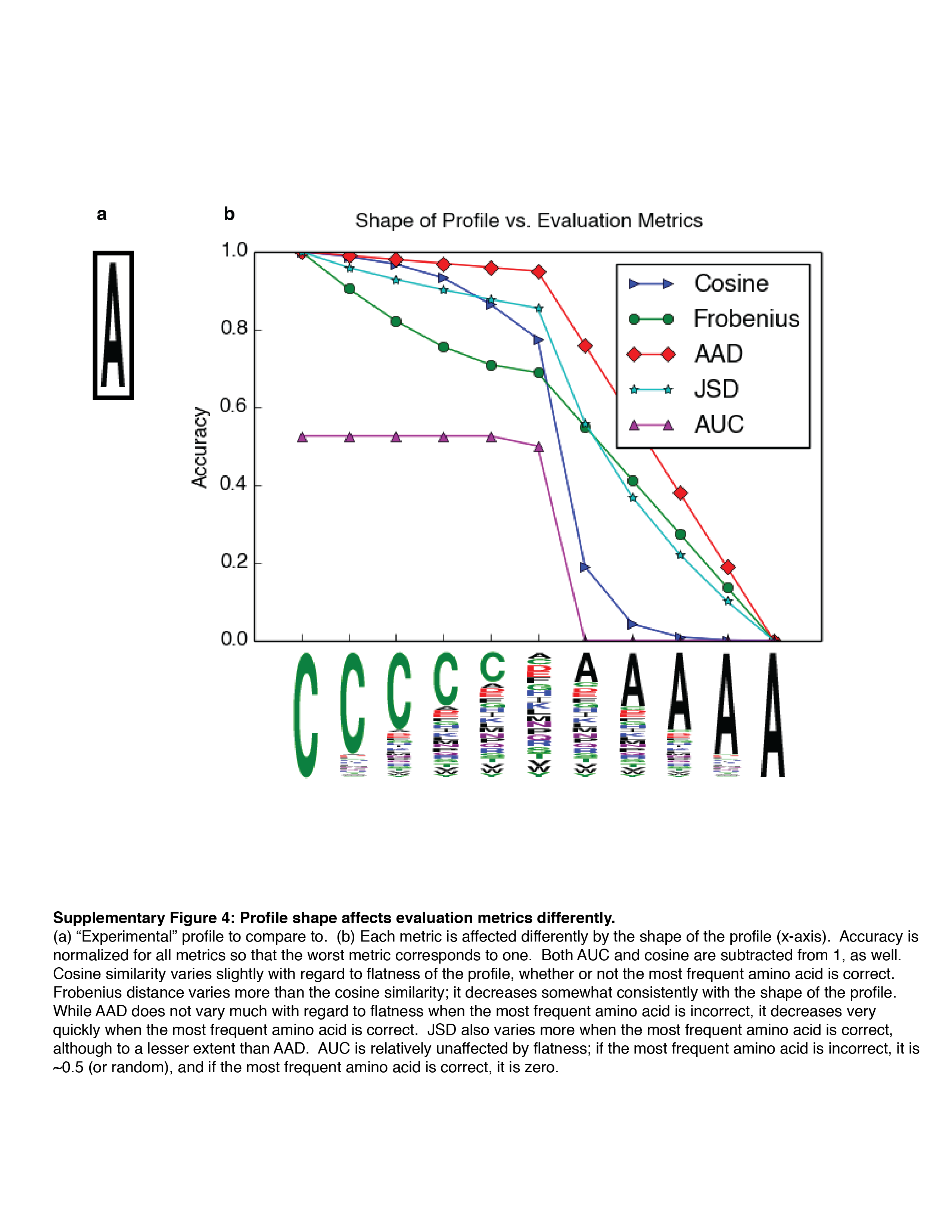

Supplement: S4 Fig — (a) “Experimental” profile to compare to. (b) Each metric is affected differently by the shape of the profile (x-axis). Accuracy is normalized for all metrics so that the worst metric corresponds to one. Both AUC and cosine are subtracted from 1, as well. Cosine similarity varies slightly with regard to flatness of the profile, whether or not the most frequent amino acid is correct. Frobenius distance varies more than the cosine similarity; it decreases somewhat consistently with the shape of the profile. While AAD does not vary much with regard to flatness when the most frequent amino acid is incorrect, it decreases very quickly when the most frequent amino acid is correct. JSD also varies more when the most frequent amino acid is correct, although to a lesser extent than AAD. AUC is relatively unaffected by flatness; if the most frequent amino acid is incorrect, it is ~0.5 (or random), and if the most frequent amino acid is correct, it is zero. (PNG) [file pcbi.1005614.s004.png]

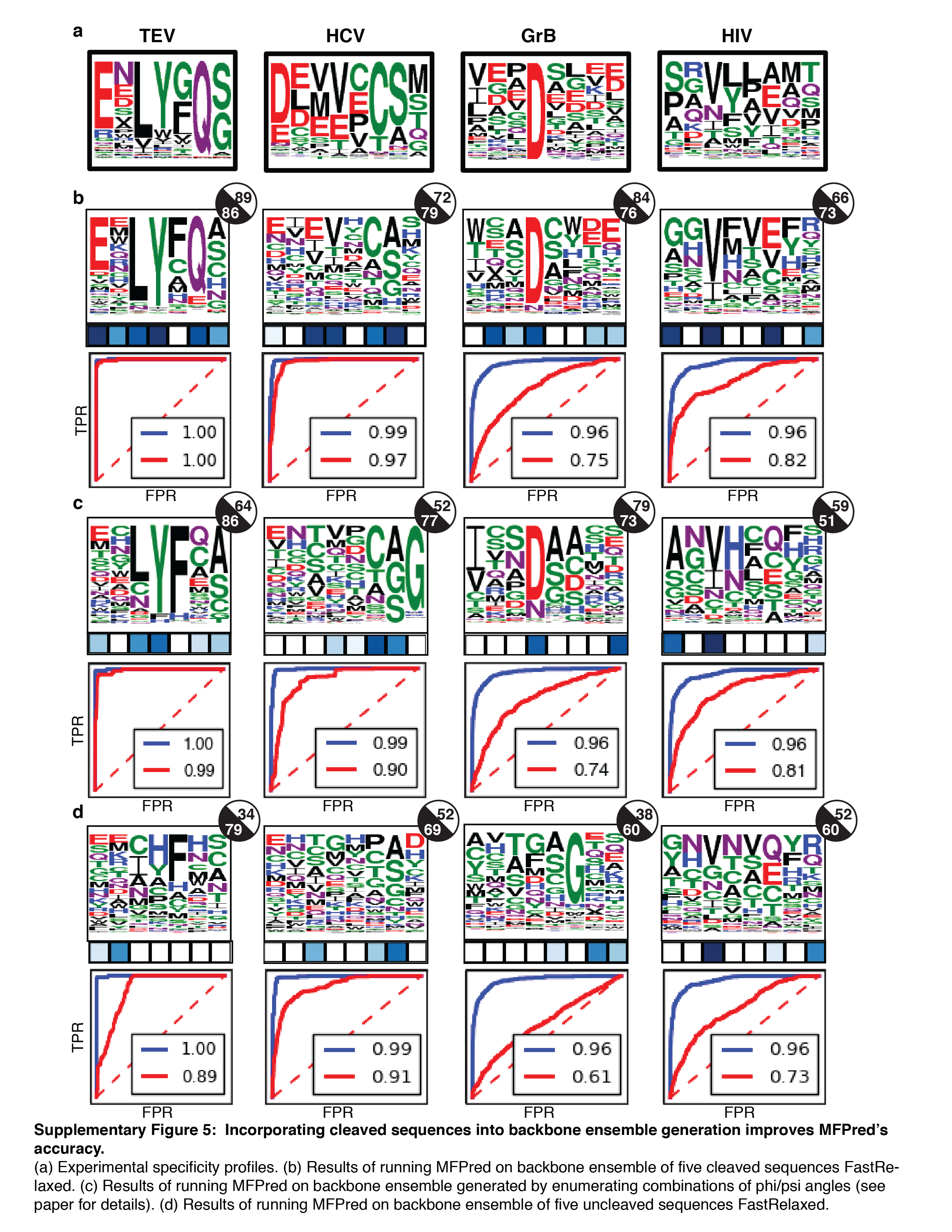

Supplement: S5 Fig — (a) Experimental specificity profiles. (b) Results of running MFPred on backbone ensemble of five cleaved sequences FastRelaxed. (c) Results of running MFPred on backbone ensemble generated by enumerating combinations of phi/psi angles (see paper for details). (d) Results of running MFPred on backbone ensemble of five uncleaved sequences FastRelaxed. (PNG) [file pcbi.1005614.s005.png]

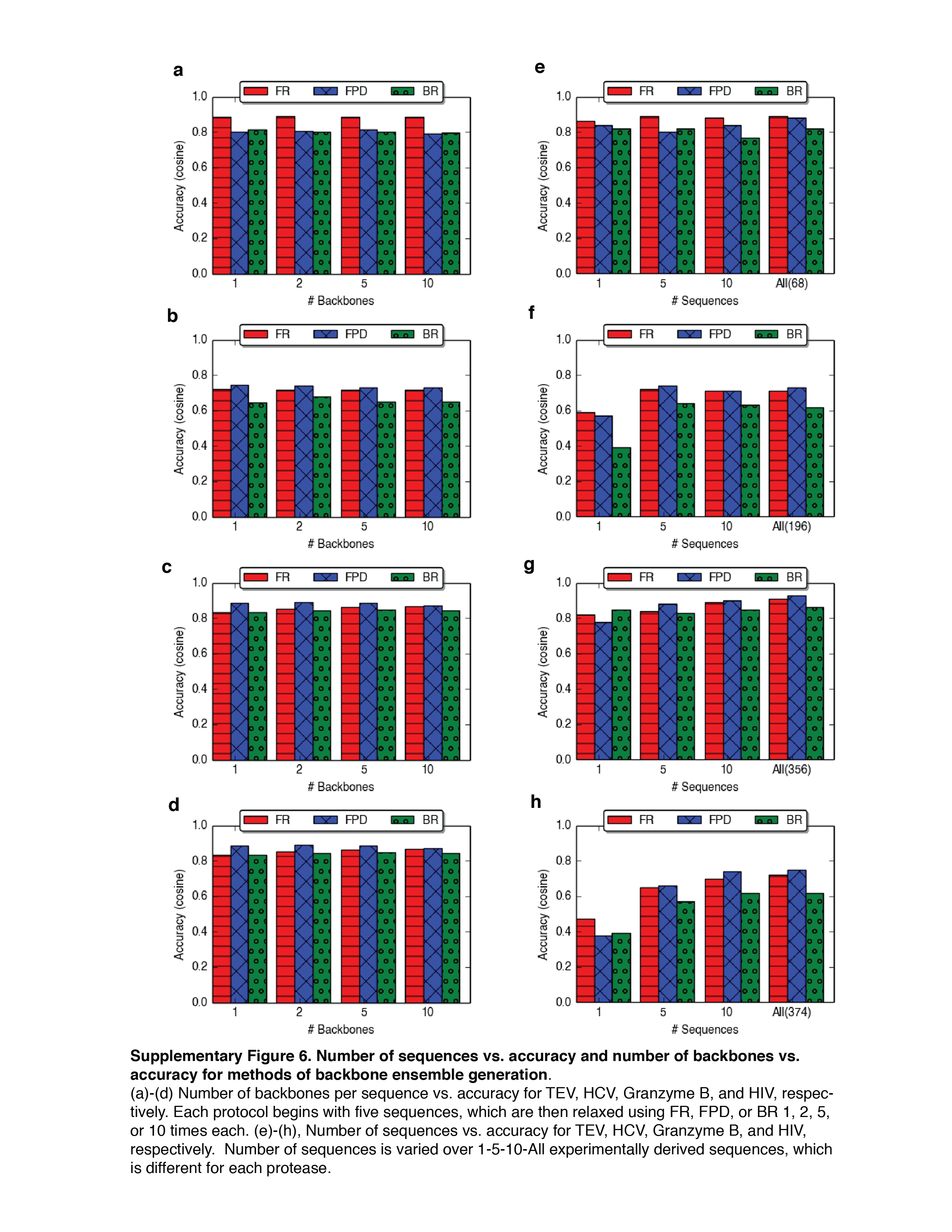

Supplement: S6 Fig — (a)-(d) Number of backbones per sequence vs. accuracy for TEV, HCV, Granzyme B, and HIV, respectively. Each protocol begins with five sequences, which are then relaxed using FR, FPD, or BR 1, 2, 5, or 10 times each. (e)-(h), Number of sequences vs. accuracy for TEV, HCV, Granzyme B, and HIV, respectively. Number of sequences is varied over 1-5-10-All experimentally derived sequences, which is different for each protease. (PNG) [file pcbi.1005614.s006.png]

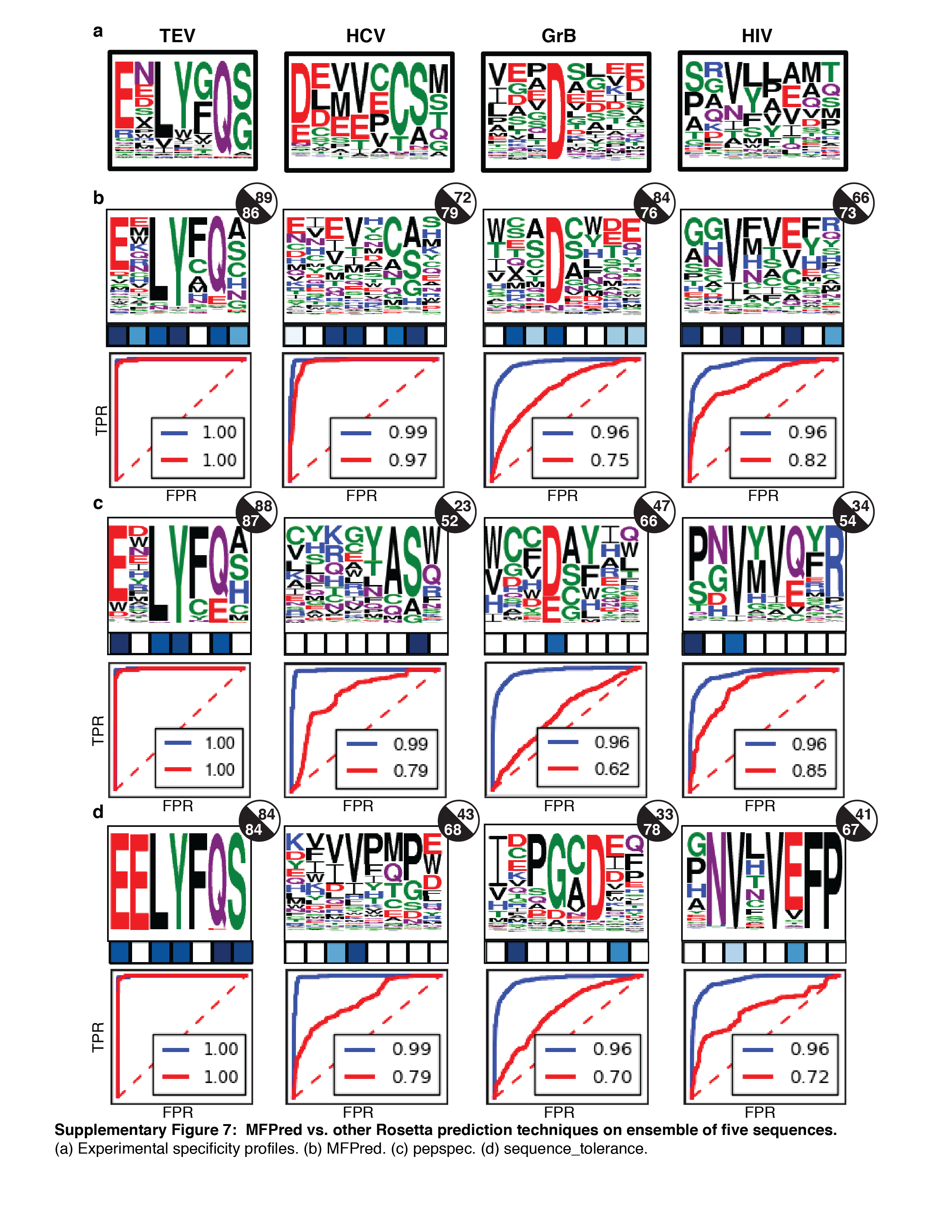

Supplement: S7 Fig — (a) Experimental specificity profiles. (b) MFPred. (c) pepspec. (d) sequence_tolerance. (PNG) [file pcbi.1005614.s007.png]

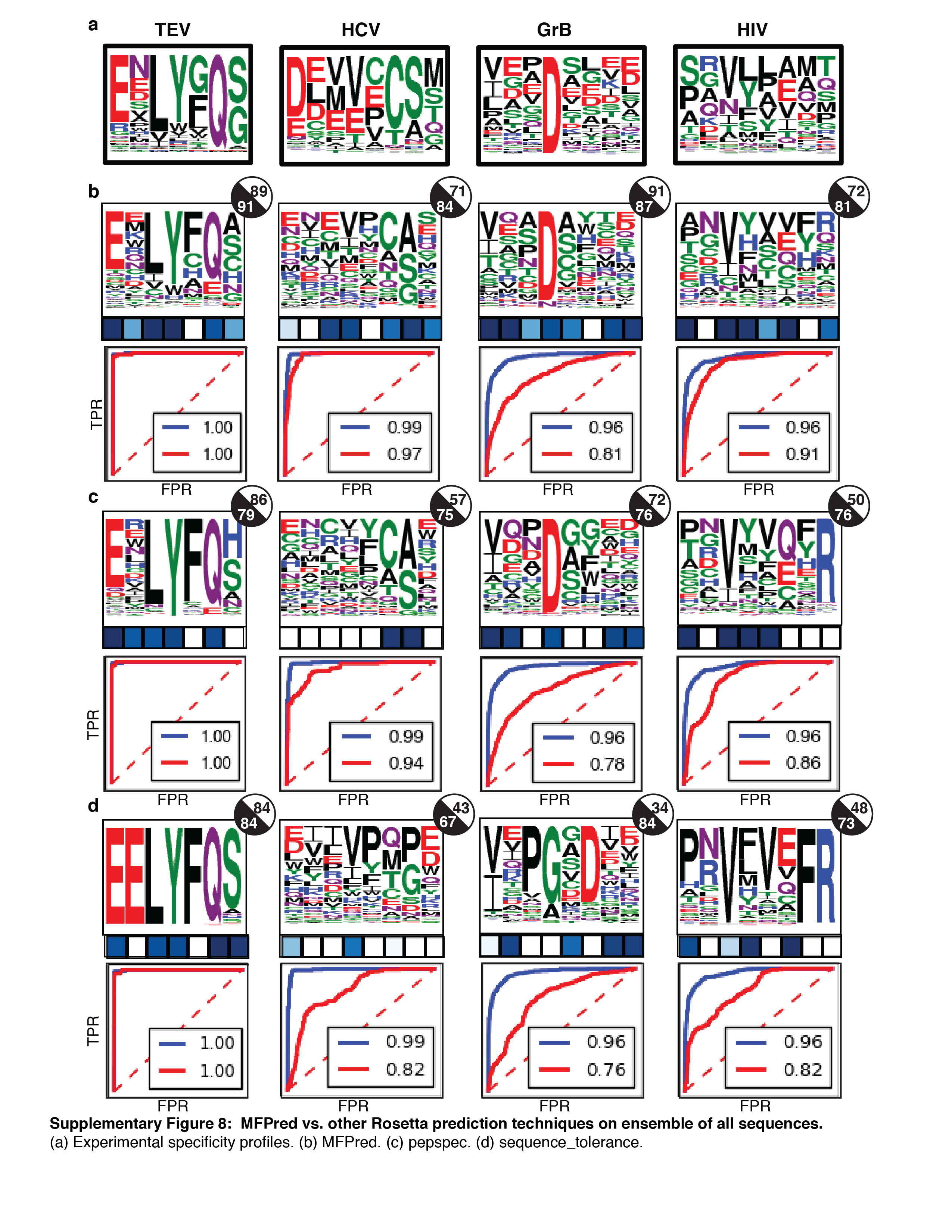

Supplement: S8 Fig — (a) Experimental specificity profiles. (b) MFPred. (c) pepspec. (d) sequence_tolerance. (PNG) [file pcbi.1005614.s008.png]

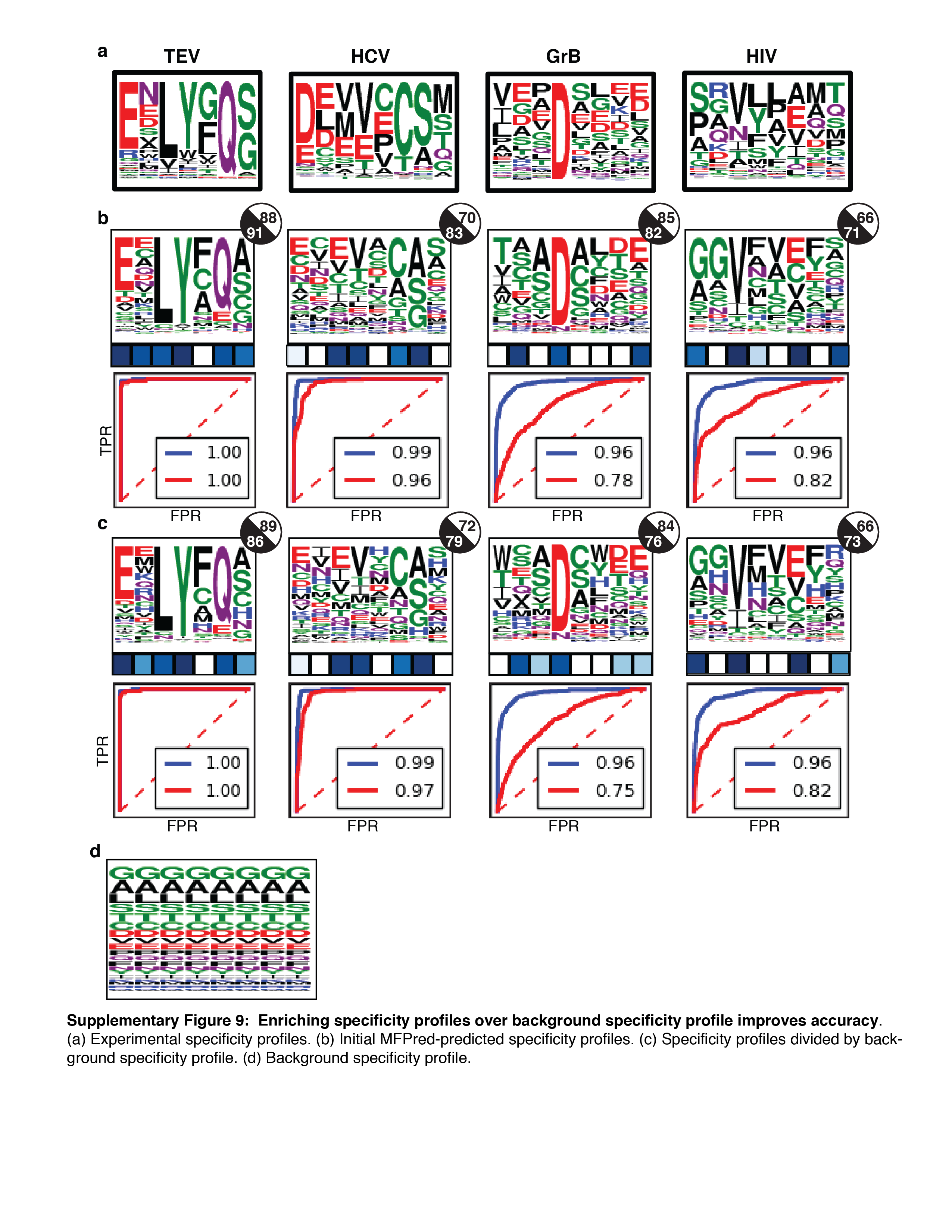

Supplement: S9 Fig — (a) Experimental specificity profiles. (b) Initial MFPred-predicted specificity profiles. (c) Specificity profiles divided by background specificity profile. (d) Background specificity profile. (PNG) [file pcbi.1005614.s009.png]

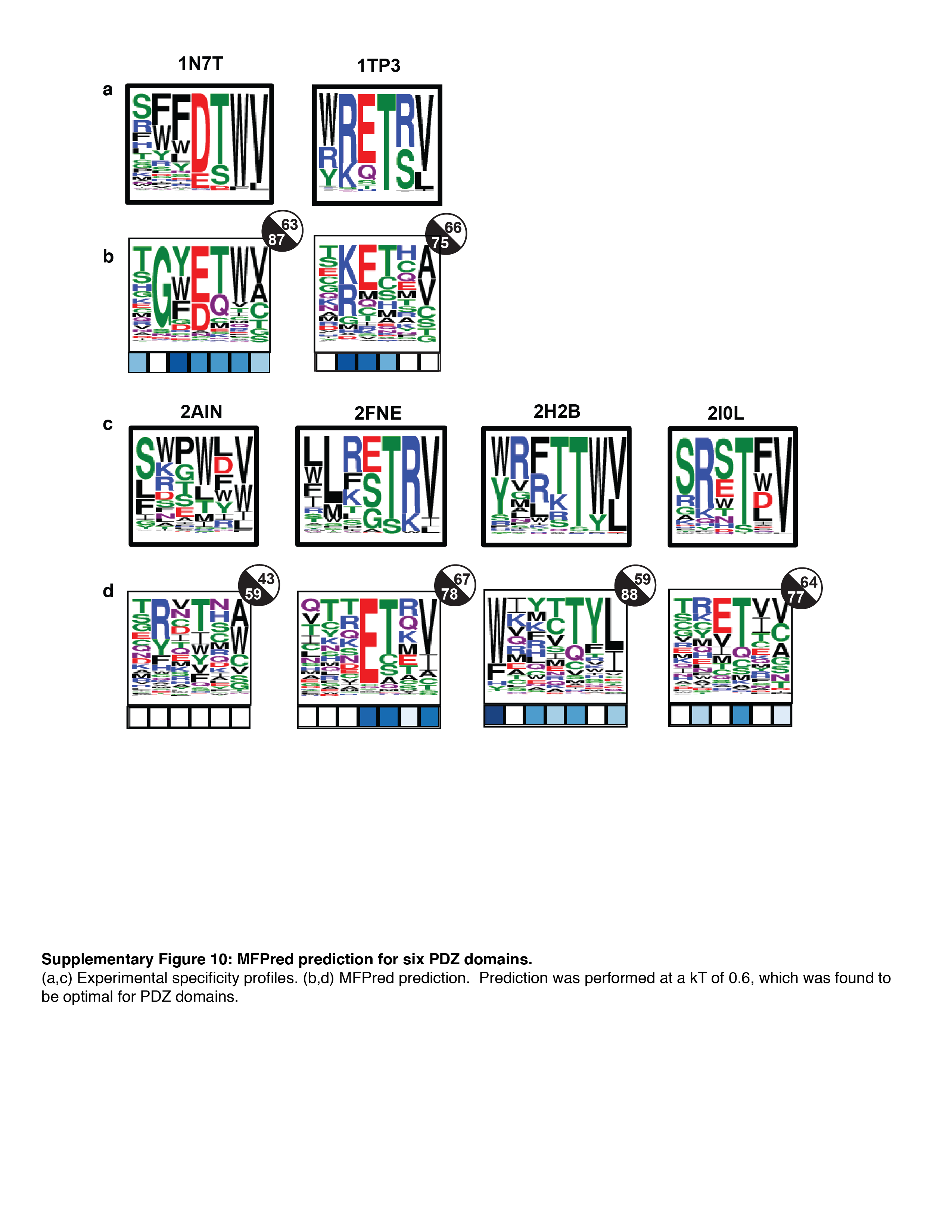

Supplement: S10 Fig — (a,c) Experimental specificity profiles. (b,d) MFPred prediction. Prediction was performed at a kT of 0.6, which was found to be optimal for PDZ domains. (PNG) [file pcbi.1005614.s010.png]

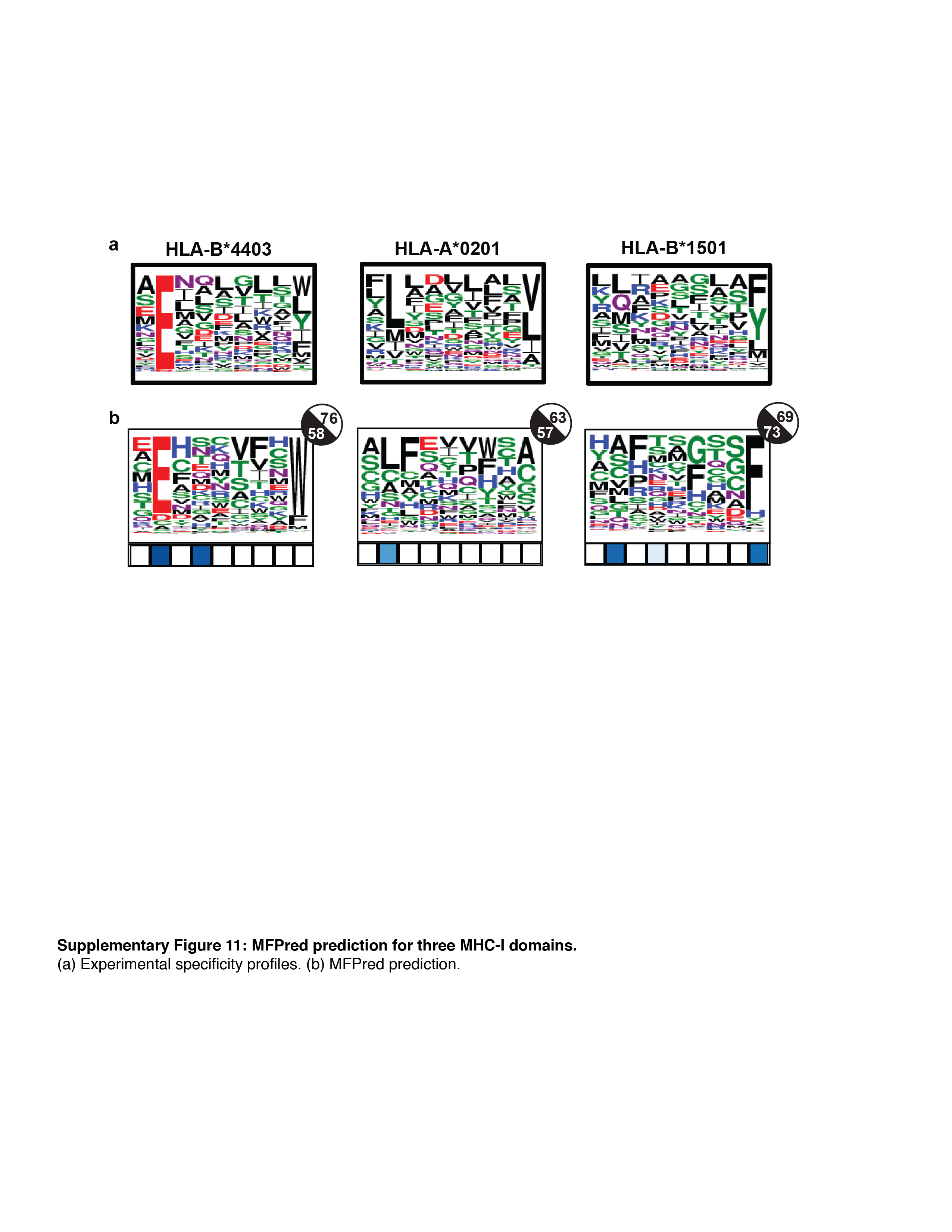

Supplement: S11 Fig — (a) Experimental specificity profiles. (b) MFPred prediction. (PNG) [file pcbi.1005614.s011.png]

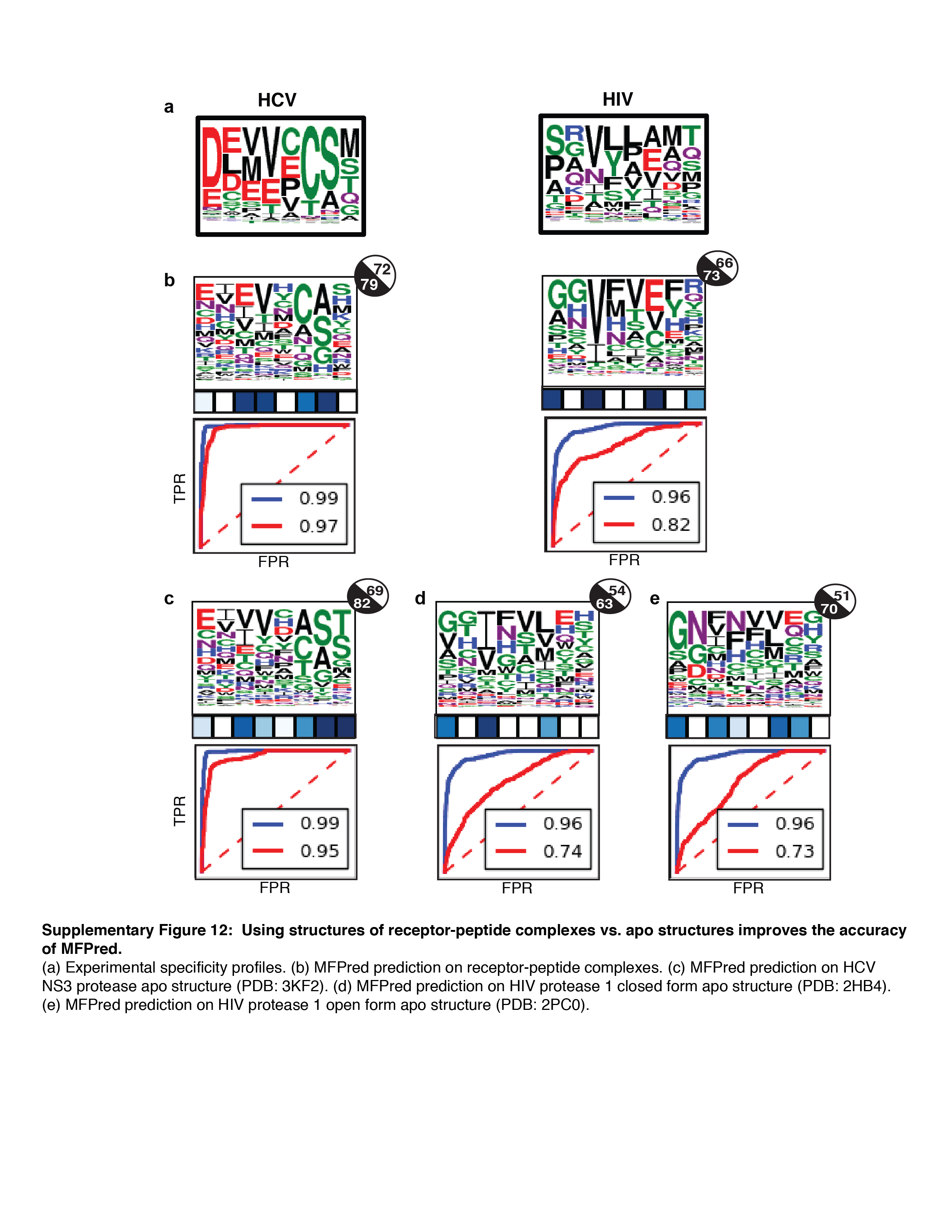

Supplement: S12 Fig — (a) Experimental specificity profiles. (b) MFPred prediction on receptor-peptide complexes. (c) MFPred prediction on HCV NS3 protease apo structure. (d) MFPred prediction on HIV protease 1 closed form apo structure. (e) MFPred prediction on HIV protease 1 open form apo structure. (PNG) [file pcbi.1005614.s012.png]
