# Supplementary material for: MFPred: Rapid and accurate prediction of protein-peptide recognition multispecificity using self-consistent mean field theory
Source: PLoS Comput Biol. 2017 Jun 26;13(6):e1005614. doi: 10.1371/journal.pcbi.1005614 (PMC5507473; doi:10.1371/journal.pcbi.1005614)
Supplement: S1 Table — (DOCX) [file pcbi.1005614.s013.docx]

**Supplementary Table 1: Details of model generation for four proteases and fourteen PRDs**

| Protein | PDB ID | Resolution | Notes |
| --- | --- | --- | --- |
| HCV NS3 **Protease** | 3M5L, 3M5N | 1.9 Å | The P’ residues of the bound peptide were built by overlaying PDB ID: 3M5N and PDB ID:3M5L (inhibitor-bound crystal structure) thus allowing us to build a complete substrate bound complex |
| HCV NS3 **Protease** (apo) | 3KF2 | 2.5 Å | PDB ID: 3KF2, the apo structure of HCV NS3 protease, was superimposed with the complex built from 3M5L and 3M5N (above) and the peptide from that model was added to the apo structure to generate the starting model. |
| TEV **Protease** | 1LVB, 1LVM | 2.2 Å | Starting model generated from PDB by reverting C151A to WT |
| Granzyme B (**Protease**) | 1FI8 | 2.2 Å | The interface of the ecotin chain in the crystal structure, spanning eight residue substrate chain was used as the starting point for further calculations |
| HIV **Protease** 1 | 1MT9 | 2.0 Å | Starting model generated by inverting D25N and V82N from crystal structure to native residue identities |
| HIV **Protease** 1 (apo) | 2HB4 | 2.15 Å | PDB ID: 2HB4, the closed-form apo structure of HIV protease-1, was superimposed with the complex built from 1MT9 (above) and the peptide from that model was added to the apo structure to generate the starting model. |
| HIV **Protease** 1 (apo) | 2PC0 | 1.4 Å | PDB ID: 2PC0, the open-form apo structure of HIV protease-1, was superimposed with the complex built from 1MT9 (above) and the peptide from that model was added to the apo structure to generate the starting model. |
| c-Crk **SH3**-N | 1CKA | 1.5 Å |  |
| cAMP-dependent PKA (**kinase**) | 1L3R | 2.0 Å |  |
| Src **SH2** | 1SPS | 2.7 Å |  |
| PSD-95 **PDZ3** | 1TP3 | 1.99 Å |  |
| NHERF-2 **PDZ2** | 2HE4 | 1.45 Å |  |
| AF-6 **PDZ** | 2AIN | (NMR) | First model in NMR ensemble was taken. |
| Erbin **PDZ** | 1N7T | (NMR) | First model in NMR ensemble was taken. |
| MPDZ-13 (**PDZ**) | 2FNE | 1.83 Å |  |
| ZO-1 **PDZ1** | 2H2B | 1.6 Å |  |
| DLG1-2 (**PDZ**) | 2I0L | 2.31 Å |  |
| HLA-A*0201 (**MHC)** | 1QSF | 2.8 Å |  |
| HLA-B*1501 (**MHC)** | 1XR9 | 1.79 Å |  |
| HLA-B*4402 (**MHC)** | 1M6O | 1.6 Å |  |
| HLA-B*4403 (**MHC)** | 1N2R | 1.7 Å |  |
