# Supplementary material for: MFPred: Rapid and accurate prediction of protein-peptide recognition multispecificity using self-consistent mean field theory
Source: PLoS Comput Biol. 2017 Jun 26;13(6):e1005614. doi: 10.1371/journal.pcbi.1005614 (PMC5507473; doi:10.1371/journal.pcbi.1005614)
Supplement: S2 Table — (DOCX) [file pcbi.1005614.s014.docx]

| **Protease** | **Method** | **#Seq** | **Cosine** | **Frob** | **AAD** | **JSD** | **AUC** | **SSAL** | **Bits** |
| --- | --- | --- | --- | --- | --- | --- | --- | --- | --- |
| **TEV** | **FR** | 1 | 0.86 | 1.06 | 0.04 | 0.22 | 0.87 | 0.00 | 0.43 |
|  |  | 5 | 0.89 | 0.85 | 0.04 | 0.21 | 0.86 | 0.00 | -0.34 |
|  |  | 10 | 0.88 | 0.86 | 0.04 | 0.20 | 0.91 | 0.00 | -0.55 |
|  |  | All (68) | 0.89 | 0.84 | 0.03 | 0.20 | 0.91 | 0.00 | -0.69 |
|  | **FPD** | 1 | 0.84 | 1.08 | 0.04 | 0.23 | 0.86 | 0.00 | 0.23 |
|  |  | 5 | 0.80 | 1.10 | 0.04 | 0.27 | 0.85 | 0.01 | -0.64 |
|  |  | 10 | 0.84 | 0.99 | 0.04 | 0.24 | 0.91 | 0.00 | -0.64 |
|  |  | All (68) | 0.88 | 0.87 | 0.04 | 0.20 | 0.91 | 0.00 | -0.72 |
|  | **BR** | 1 | 0.82 | 1.11 | 0.04 | 0.25 | 0.84 | 0.00 | -0.06 |
|  |  | 5 | 0.82 | 1.06 | 0.05 | 0.26 | 0.87 | 0.00 | -0.70 |
|  |  | 10 | 0.77 | 1.17 | 0.05 | 0.29 | 0.89 | 0.00 | -0.91 |
|  |  | All (68) | 0.82 | 1.06 | 0.05 | 0.27 | 0.89 | 0.00 | -0.87 |
| **HCV** | **FR** | 1 | 0.59 | 1.37 | 0.06 | 0.35 | 0.77 | 0.08 | -0.51 |
|  |  | 5 | 0.72 | 1.13 | 0.05 | 0.31 | 0.79 | 0.02 | -1.28 |
|  |  | 10 | 0.71 | 1.15 | 0.05 | 0.30 | 0.82 | 0.02 | -1.28 |
|  |  | All (196) | 0.71 | 1.14 | 0.05 | 0.29 | 0.84 | 0.02 | -1.29 |
|  | **FPD** | 1 | 0.57 | 1.45 | 0.06 | 0.35 | 0.76 | 0.09 | -0.39 |
|  |  | 5 | 0.74 | 1.10 | 0.05 | 0.30 | 0.83 | 0.02 | -1.29 |
|  |  | 10 | 0.71 | 1.14 | 0.05 | 0.30 | 0.80 | 0.01 | -1.29 |
|  |  | All (196) | 0.73 | 1.12 | 0.05 | 0.28 | 0.87 | 0.01 | -1.35 |
|  | **BR** | 1 | 0.39 | 1.67 | 0.06 | 0.44 | 0.69 | 0.17 | -0.83 |
|  |  | 5 | 0.64 | 1.23 | 0.05 | 0.32 | 0.80 | 0.05 | -1.20 |
|  |  | 10 | 0.63 | 1.25 | 0.06 | 0.32 | 0.81 | 0.04 | -1.22 |
|  |  | All (196) | 0.62 | 1.26 | 0.05 | 0.32 | 0.81 | 0.05 | -1.31 |
| **GrB** | **FR** | 1 | 0.82 | 0.85 | 0.04 | 0.23 | 0.71 | 0.20 | 0.60 |
|  |  | 5 | 0.84 | 0.73 | 0.04 | 0.20 | 0.76 | 0.21 | 0.07 |
|  |  | 10 | 0.89 | 0.60 | 0.03 | 0.17 | 0.80 | 0.17 | 0.06 |
|  |  | All (356) | 0.91 | 0.53 | 0.03 | 0.13 | 0.87 | 0.15 | -0.08 |
|  | **FPD** | 1 | 0.78 | 1.04 | 0.04 | 0.25 | 0.72 | 0.19 | 0.83 |
|  |  | 5 | 0.88 | 0.62 | 0.03 | 0.17 | 0.76 | 0.18 | 0.10 |
|  |  | 10 | 0.90 | 0.59 | 0.03 | 0.15 | 0.80 | 0.17 | 0.02 |
|  |  | All (356) | 0.93 | 0.49 | 0.03 | 0.11 | 0.83 | 0.13 | -0.08 |
|  | **BR** | 1 | 0.85 | 0.74 | 0.04 | 0.22 | 0.71 | 0.19 | 0.38 |
|  |  | 5 | 0.83 | 0.74 | 0.04 | 0.20 | 0.71 | 0.22 | 0.14 |
|  |  | 10 | 0.85 | 0.70 | 0.04 | 0.19 | 0.72 | 0.22 | 0.09 |
|  |  | All (356) | 0.86 | 0.68 | 0.04 | 0.18 | 0.72 | 0.21 | 0.08 |
| **HIV** | **FR** | 1 | 0.47 | 1.55 | 0.06 | 0.42 | 0.66 | 0.17 | 0.96 |
|  |  | 5 | 0.65 | 0.96 | 0.05 | 0.27 | 0.73 | 0.14 | -0.01 |
|  |  | 10 | 0.70 | 0.88 | 0.04 | 0.23 | 0.78 | 0.08 | -0.04 |
|  |  | All (374) | 0.72 | 0.82 | 0.04 | 0.21 | 0.81 | 0.05 | -0.21 |
|  | **FPD** | 1 | 0.38 | 1.78 | 0.07 | 0.47 | 0.69 | 0.22 | 1.22 |
|  |  | 5 | 0.66 | 0.96 | 0.05 | 0.28 | 0.70 | 0.13 | -0.04 |
|  |  | 10 | 0.74 | 0.81 | 0.04 | 0.22 | 0.78 | 0.07 | -0.18 |
|  |  | All (374) | 0.75 | 0.77 | 0.04 | 0.19 | 0.83 | 0.05 | -0.32 |
|  | **BR** | 1 | 0.39 | 1.48 | 0.06 | 0.41 | 0.67 | 0.23 | 0.47 |
|  |  | 5 | 0.57 | 1.06 | 0.05 | 0.30 | 0.74 | 0.15 | -0.04 |
|  |  | 10 | 0.62 | 0.98 | 0.05 | 0.27 | 0.73 | 0.14 | -0.11 |
|  |  | All (374) | 0.62 | 0.96 | 0.05 | 0.27 | 0.73 | 0.11 | -0.16 |
| Most Similar | |  | 1.00 | 0.00 | 0.00 | 0.00 | 1.00 | 0.00 | 0.00 |
| Most Different | |  | 0.00 | √(2n)^1^ | 0.06 | 1.00 | 0.00 | 1.00 | 4.32 |

^1^n refers to the number of positions in the profile

**Supplementary Table 2. Results of MFPred on different backbone ensembles.**
