# Supplementary material for: MFPred: Rapid and accurate prediction of protein-peptide recognition multispecificity using self-consistent mean field theory
Source: PLoS Comput Biol. 2017 Jun 26;13(6):e1005614. doi: 10.1371/journal.pcbi.1005614 (PMC5507473; doi:10.1371/journal.pcbi.1005614)
Supplement: S3 Table — (DOCX) [file pcbi.1005614.s015.docx]

| **Protease** | **Method** | **Cosine** | **Frob** | **AAD** | **JSD** | **AUC** | **SSAL** | **Bits** |
| --- | --- | --- | --- | --- | --- | --- | --- | --- |
| **TEV** | **Current** | 0.89 | 0.85 | 0.04 | 0.21 | 0.86 | 0.00 | -0.34 |
|  | **Dun02** | 0.86 | 0.97 | 0.04 | 0.24 | 0.86 | 0.00 | -0.14 |
|  | **Ex1aro,ex2aro** | 0.89 | 0.85 | 0.04 | 0.21 | 0.86 | 0.00 | -0.34 |
|  | **Ex3,ex4** | 0.88 | 0.87 | 0.04 | 0.22 | 0.86 | 0.00 | -0.38 |
|  | **No input sc** | 0.88 | 0.88 | 0.04 | 0.22 | 0.86 | 0.00 | -0.46 |
|  | **Pack prot 4** | 0.81 | 1.07 | 0.04 | 0.25 | 0.90 | 0.00 | -0.56 |
|  | **Pack prot 6** | 0.81 | 1.07 | 0.04 | 0.25 | 0.91 | 0.00 | -0.59 |
|  | **Pack prot 8** | 0.81 | 1.07 | 0.04 | 0.25 | 0.91 | 0.00 | -0.60 |
| **HCV** | **Current** | 0.72 | 1.13 | 0.05 | 0.31 | 0.79 | 0.02 | -1.28 |
|  | **Dun02** | 0.64 | 1.24 | 0.06 | 0.35 | 0.78 | 0.02 | -1.19 |
|  | **Ex1aro,ex2aro** | 0.72 | 1.13 | 0.05 | 0.31 | 0.79 | 0.02 | -1.28 |
|  | **Ex3,ex4** | 0.71 | 1.14 | 0.05 | 0.31 | 0.77 | 0.03 | -1.27 |
|  | **No input sc** | 0.71 | 1.15 | 0.05 | 0.31 | 0.78 | 0.02 | -1.29 |
|  | **Pack prot 4** | 0.67 | 1.20 | 0.06 | 0.33 | 0.73 | 0.04 | -1.21 |
|  | **Pack prot 6** | 0.67 | 1.20 | 0.06 | 0.33 | 0.74 | 0.04 | -1.21 |
|  | **Pack prot 8** | 0.67 | 1.20 | 0.06 | 0.33 | 0.74 | 0.04 | -1.20 |
| **GrB** | **Current** | 0.84 | 0.73 | 0.04 | 0.20 | 0.76 | 0.21 | 0.07 |
|  | **Dun02** | 0.82 | 0.78 | 0.04 | 0.23 | 0.79 | 0.22 | 0.21 |
|  | **Ex1aro,ex2aro** | 0.84 | 0.73 | 0.04 | 0.20 | 0.76 | 0.21 | 0.07 |
|  | **Ex3,ex4** | 0.84 | 0.73 | 0.04 | 0.20 | 0.76 | 0.21 | 0.08 |
|  | **No input sc** | 0.84 | 0.73 | 0.04 | 0.20 | 0.75 | 0.22 | 0.06 |
|  | **Pack prot 4** | 0.81 | 0.80 | 0.04 | 0.23 | 0.77 | 0.25 | 0.22 |
|  | **Pack prot 6** | 0.80 | 0.82 | 0.04 | 0.23 | 0.75 | 0.26 | 0.18 |
|  | **Pack prot 8** | 0.81 | 0.80 | 0.04 | 0.23 | 0.76 | 0.25 | 0.21 |
| **HIV** | **Current** | 0.65 | 0.96 | 0.05 | 0.27 | 0.73 | 0.14 | -0.01 |
|  | **Dun02** | 0.59 | 1.08 | 0.05 | 0.32 | 0.68 | 0.14 | 0.10 |
|  | **Ex1aro,ex2aro** | 0.65 | 0.96 | 0.05 | 0.27 | 0.73 | 0.14 | -0.01 |
|  | **Ex3,ex4** | 0.65 | 0.97 | 0.05 | 0.27 | 0.71 | 0.14 | -0.01 |
|  | **No input sc** | 0.63 | 0.98 | 0.05 | 0.28 | 0.70 | 0.15 | -0.06 |
|  | **Pack prot 4** | 0.63 | 1.01 | 0.05 | 0.30 | 0.71 | 0.14 | 0.08 |
|  | **Pack prot 6** | 0.61 | 1.04 | 0.05 | 0.32 | 0.71 | 0.15 | 0.11 |
|  | **Pack prot 8** | 0.60 | 1.05 | 0.05 | 0.31 | 0.69 | 0.15 | 0.05 |
| Most Similar |  | 1.00 | 0.00 | 0.00 | 0.00 | 1.00 | 0.00 | 0.00 |
| Most Different | | 0.00 | √(2n)^1^ | 0.06 | 1.00 | 0.00 | 1.00 | 4.32 |

**Supplementary Table 3. Effect of various Rosetta settings on MFPred predictions on five sequence backbones.**

^1^n refers to the number of positions in the profile
