# Supplementary material for: MFPred: Rapid and accurate prediction of protein-peptide recognition multispecificity using self-consistent mean field theory
Source: PLoS Comput Biol. 2017 Jun 26;13(6):e1005614. doi: 10.1371/journal.pcbi.1005614 (PMC5507473; doi:10.1371/journal.pcbi.1005614)
Supplement: S1 Note — (DOCX) [file pcbi.1005614.s016.docx]

**Supplementary Note 1**

We used several metrics and distances to evaluate specificity profile predictions. The Frobenius distance is defined as:

$$Frobenius(E,P)=\sqrt{\sum_{i=1}^{N} (E_{i}-P_{i})^{2}}$$

where E is a vector of experimentally determined amino acid frequencies and P is a vector of predicted frequencies. To calculate the Frobenius distance of the entire profile, we simply flattened the experimental and predicted profiles into one vector each. Two identical probability distributions have a Frobenius distance of 0, while two most divergent distributions have a Frobenius distance of (2n)^1/2^, where n is equal to the number of positions in the profile.

The Average Absolute Distance (AAD) is defined as:

$$AAD(E,P)=\frac{1}{N}\sum_{i=1}^{N} \left| E_{i}-P_{i} \right|$$

Again, to calculate the AAD of the entire profile, we flattened each profile to a single vector. AAD ranges between 0 to 1, with 0 as the best score and 1 as the worst score. According to Smith and Kortemme, an AAD of less than 6% (or 0.06) is considered to be a good prediction.

The cosine similarity is defined as:

$$Cosine(E,P)=\frac{\sum_{i=1}^{N} E_{i}P_{i}}{\sqrt{\sum_{i=1}^{N} E_{i}^{2}}\sqrt{\sum_{i=1}^{N} P_{i}^{2}}}$$

We flattened each profile to a single vector. Two identical specificity profiles have a cosine distance of 1 whereas two most divergent profiles have a similarity of 0.

Jensen-Shannon Divergence (JSD) is defined as:

$$JSD\left( E,P \right)=H\left( \sum_{i=1}^{N} 0.5E_{i}+0.5P_{i} \right)- 0.5\sum_{i=1}^{N} H\left( E_{i} \right)-0.5\sum_{i=1}^{N} H\left( P_{i} \right)$$

where H is Shannon entropy, defined as:

$$H\left( E \right)=-\sum_{i=1}^{N} E_{i}\log_{2} E_{i}$$

We calculated the JSD of the entire profile by averaging the JSD of each vector (or position) in the profile. A JSD of zero denotes two identical profiles, whereas a JSD of 1 denotes two entirely divergent profiles. While JSD is not considered a proper metric, it does provide information regarding how divergent two profiles are.

Area under the ROC curve, or AUC, as developed by Smith and Kortemme [1], is another measure that we used to evaluate the profiles. We plotted an ROC curve for each predicted profile based on how well the most frequent experimental amino acids (defined as > 10%) are recapitulated in the predicted profile. We then calculated the area under the curve, which denotes the probability that the predicted profile ranks a positive amino acid as higher than a negative amino acid. An AUC of 1 represents a perfect prediction, while an AUC of 0.5 is equivalent to a random prediction.

Last, we developed a new distance, referred to as the Score-Sequence AUC Loss (SSAL). This distance also takes advantage of an ROC curve, although this one is slightly different. We use the experimental profile to generate a score for each cleaved and uncleaved sequence by taking the sum of the probabilities of each amino acid in the sequence occurring at its position:

$$Score\left( S \right)=\sum_{i=1}^{len(S)} E_{i}(S_{i})$$

We then plot an ROC curve that demonstrates how well the scores rank the cleaved vs. uncleaved sequences and calculate its AUC. We repeat the entire process with the predicted profile, and then subtract the predicted ROC-AUC from the experimental ROC-AUC. The result is the SSAL, which denotes how well the predicted profile differentiates between cleaved/uncleaved sequences vs. the experimental profile.

In order to transform the values of the distances to *p*-values, we generated 100,000 random profiles by randomly sampling columns of our protease and PRD experimental profile library and randomly shuffling the amino acid identity of their frequencies so as to generate profiles with similar information content. We then calculated their per-column and overall distance from each experimental profile for each of the six measures. The ranking of a given predicted profile distance value in its given distance list was then used to find the *p*-value.

**References**

1. Smith CA, Kortemme T. Structure-Based Prediction of the Peptide Sequence Space Recognized by Natural and Synthetic PDZ Domains. J Mol Biol. 2010;402(2):460–74.
