# Supplementary material for: MFPred: Rapid and accurate prediction of protein-peptide recognition multispecificity using self-consistent mean field theory
Source: PLoS Comput Biol. 2017 Jun 26;13(6):e1005614. doi: 10.1371/journal.pcbi.1005614 (PMC5507473; doi:10.1371/journal.pcbi.1005614)
Supplement: S2 Note — (DOCX) [file pcbi.1005614.s017.docx]

**Running Entire MFPred pipeline:**

**Inputs**

- Crystallographic pdb of protein-peptide complex
- List of five substrate sequences to thread on

**Process**

1. [Initial Relax](#init_relax)
   1. Run on initial crystallographic pdb to get rid of internal clashes
2. [Thread Peptide-FastRelax](#thread_pept)
   1. Run this step for each substrate sequence
3. [MFPred](#MFPred)
   1. Choose the lowest-scoring pdb from 2a for each substrate sequence and use a list of paths to these pdbs as the input for MFPred
4. [Distances.py](#distances) (optional)

**Outputs**

- Transfac file for each pdb and averaged transfac file
- Distance file (distances per-column and overall)

**Initial Relax**

**Inputs**

1. **<PATH_TO_XTAL_PDB>** **Crystallographic pdb of protein-peptide complex**

Retrieve from pdb

1. **<PATH_TO_ENZDES_CSTFILE> (for proteases only)**

Generate yourself based on protease catalytic geometry

1. **<PATH_TO_COO_CSTFILE> (for proteases only)**

Use a modified version of sidechain_cst_3.py (at /source/src/apps/public/relax_w_allatom_cst/sidechain_cst_3.py in the Rosetta source code) to generate constraints with settings of 0.1 and 0.5 on the protease atoms.

1. **<RESFILE>**

NATRO all, NATAA peptide residues

1. **<XML_FILE>**

*Sample xml:*

<ROSETTASCRIPTS>

<SCOREFXNS>

<ScoreFunction name="myscore" weights="<SCORE_FUNCTION>".wts/>

</SCOREFXNS>

<TASKOPERATIONS>

<ProteinInterfaceDesign design_chain2="0" modify_after_jump="1" name="pido"/>

<InitializeFromCommandline name="init"/>

<ReadResfile name="rrf"/>

</TASKOPERATIONS>

<FILTERS/>

<MOVERS>

<AddOrRemoveMatchCsts cst_instruction="add_new" name="cstadd"/>

<FastRelax name="fastrelax" repeats="8" scorefxn="myscore" task_operations="pido,init">

<MoveMap name="mm">

<Chain bb="1" chi="1" number="2"/>

<Chain bb="1" chi="1" number="1"/>

<Jump number="1" setting="1"/>

</MoveMap>

</FastRelax>

<TaskAwareMinMover bb="0" chi="1" jump="0" name="min_pro" scorefxn="myscore" task_operations="rrf"/>

<PackRotamersMover name="repack" task_operations="rrf"/>

<ConstraintSetMover name="protease_cst"/>

</MOVERS>

<APPLY_TO_POSE/>

<PROTOCOLS>

<Add mover_name="protease_cst"/>

<Add mover_name="repack"/>

<Add mover_name="min_pro"/>

<Add mover_name="cstadd"/>

<Add mover_name="fastrelax"/>

</PROTOCOLS>

</ROSETTASCRIPTS>

1. **<PATH_TO_FLAGS>**

-mute core.io.database

-packing::use_input_sc

-packing::extrachi_cutoff 1

-packing::ex1

-packing::ex2

-linmem_ig 10

-out:file::output_virtual

**Process**

Run on initial crystallographic pdb to get rid of internal clashes.

*Command Line:*

<ROSETTA_BIN>rosetta_scripts.static.linuxgccrelease -jd2:ntrials 1 -nstruct 1000 -parser:protocol <XML_FILE> -database <ROSETTA_DB> -s <PATH_TO_XTAL_PDB> -run:preserve_header -enzdes::cstfile <PATH_TO_ENZDES_CSTFILE> -constraints:cst_file <PATH_TO_COO_CSTFILE> -resfile <PATH_TO_RESFILE> @<PATH_TO_FLAGS>

**Outputs**

1000 “relaxed” pdb files. Use lowest scoring pdb file as input for the next step.

**Remarks**

*Differences between protease and PRD:*

*Protease:*

command line includes: -enzdes::cstfile <PATH_TO_ENZDES_CSTFILE> -constraints:cst_file <PATH_TO_COO_CSTFILE>

<SCORE_FUNCTION>: talaris2013_cst

*PRD:*

command line does not include constraint parameters

<SCORE_FUNCTION>: talaris2013

**Thread Peptide-FastRelax**

**Inputs**

1. **<STARTING_RELAXED_MODEL> Lowest scoring pdb from** [**Initial Relax**](#init_relax) **step.**
2. **<PATH_TO_ENZDES_CSTFILE> (for proteases only)**

Generate yourself based on protease catalytic geometry

1. **<RESFILE>**

NATRO all, NATAA peptide residues

1. **<XML_FILE>**

*Sample xml:*

<ROSETTASCRIPTS>

<SCOREFXNS>

</SCOREFXNS>

<TASKOPERATIONS>

<ProteinInterfaceDesign name="pido" design_chain2="0" modify_after_jump="1" />

<InitializeFromCommandline name="init"/>

<ReadResfile name="rrf" filename=<RESFILE> />

</TASKOPERATIONS>

<FILTERS>

</FILTERS>

<MOVERS>

<MutateResidue name="mut1" target="<PEPT_RES1>" new_res="DM1"/>

<MutateResidue name="mut2" target="<PEPT_RES2>" new_res="DM2"/>

<MutateResidue name="mut3" target="<PEPT_RES3>" new_res="DM3"/>

<MutateResidue name="mut4" target="<PEPT_RES4>" new_res="DM4"/>

<MutateResidue name="mut5" target="<PEPT_RES5>" new_res="DM5"/>

<MutateResidue name="mut6" target="<PEPT_RES6>" new_res="DM6"/>

<MutateResidue name="mut7" target="<PEPT_RES7>" new_res="DM7"/>

<AddOrRemoveMatchCsts name="cstadd" cst_instruction="add_new" />

<FastRelax name="fastrelax" repeats="8" task_operations="pido,init">

<MoveMap name="mm">

<Chain number="2" chi="1" bb="1"/>

<Chain number="1" chi="1" bb="0"/>

<Jump number="1" setting="1"/>

</MoveMap>

</FastRelax>

<PackRotamersMover name="repack" task_operations="rrf"/>

</MOVERS>

<APPLY_TO_POSE>

</APPLY_TO_POSE>

<PROTOCOLS>

<Add mover_name="mut1"/>

<Add mover_name="mut2"/>

<Add mover_name="mut3"/>

<Add mover_name="mut4"/>

<Add mover_name="mut5"/>

<Add mover_name="mut6"/>

<Add mover_name="mut7"/>

<Add mover_name="cstadd"/>

<Add mover_name="repack"/>

<Add mover_name="fastrelax"/>

</PROTOCOLS>

</ROSETTASCRIPTS>

1. **<PATH_TO_FLAGS>**

-mute core.io.database

-packing::use_input_sc

-packing::extrachi_cutoff 1

-packing::ex1

-packing::ex2

-linmem_ig 10

-out:file::output_virtual

**Process**

Run on lowest scoring relaxed pdb from [Initial Relax](#init_relax) one time per substrate sequence. Substitute your peptide sequence for <PEPT_RES1>, etc. in xml script. Add more <MutateResidue> movers as needed. Generates 10 relaxed protease-peptide complexes with that substrate sequence threaded on. Select lowest-scoring complex from these 10 complexes for [MFPred](#MFPred) step.

*Command Line:*

<ROSETTA_BIN>rosetta_scripts.static.linuxgccrelease -nstruct 10 -jd2:ntrials 1 -parser:protocol <XML_FILE> -database /home/arubenstein/Rosetta/main/database/ <CONST_ARG> -s <STARTING_RELAXED_MODEL> -run:preserve_header -overwrite @<PATH_TO_FLAGS> -score:weights <SCORE_FUNCTION>

**Outputs**

10 “relaxed” pdb files. Use lowest scoring pdb file as input for the next step.

**Remarks**

*Differences between protease and PRD:*

*Protease*:

command line includes <CONST_ARG>: -enzdes::cstfile <PATH_TO_ENZDES_CSTFILE>

<SCORE_FUNCTION>: talaris2013_cst

*PRD:*

command line does not include constraint parameters

<SCORE_FUNCTION>: talaris2013

**MFPred**

**Inputs**

1. **<PATH_TO_INPUT_PDB> Lowest scoring pdb from** [**Initial Relax**](#init_relax) **step.**
2. **<LIST_PDB_COMPLEXES>**

List of paths to lowest-scoring pdbs for each of the [Thread Peptide](#thread_pept) runs in the previous step.

1. **<RESFILE>**

NATRO all, NATAA peptide residues that should not be designed (flanking residues), ALLAA peptide residues for which a specificity profile should be predicted.

1. **<XML_FILE>**

*Sample xml:*

<ROSETTASCRIPTS>

<TASKOPERATIONS>

<InitializeFromCommandline name="init" />

<ReadResfile name="rrf" />

</TASKOPERATIONS>

<SCOREFXNS>

</SCOREFXNS>

<FILTERS>

</FILTERS>

<MOVERS>

<GenMeanFieldMover name="boltz" threshold="5" lambda_memory="0.5" tolerance="0.0001" temperature="0.8" task_operations="rrf,init"/>

</MOVERS>

<APPLY_TO_POSE>

</APPLY_TO_POSE>

<PROTOCOLS>

<Add mover_name="boltz"/>

</PROTOCOLS>

</ROSETTASCRIPTS>

1. **<PATH_TO_FLAGS>**

-mute core.io.database

-packing::use_input_sc

-packing::extrachi_cutoff 1

-packing::ex1

-packing::ex2

-out:file::output_virtual

1. **<EXPT_SPEC_PROFILE> (optional)**

Path to known (experimentally-derived) specificity profile. MFPred protocol will output certain distances from this profile in the log if this parameter is given.

1. **<ROT_NORM_PARAM> (optional)**

This is the γ parameter described in the paper. The default is 0.8.

1. **<BB_AVERAGE_PARAM>**

This is the γ parameter described in the paper. The default is 0.8.

**Process**

Run on backbone ensemble as generated in [Thread Peptide](#thread_pept) step. Runs MFPred algorithm on residues that are designated as packed/designed in the TaskOperations.

*Command Line:*

<ROSETTA_BIN>rosetta_scripts.static.linuxgccrelease -database <ROSETTA_DB> -parser:protocol <XML_FILE> -s <PATH_TO_INPUT_PDB> -rot_norm_weight <ROT_NORM_PARAM> -bb_average_weight <BB_AVERAGE_PARAM> -spec_profile <EXPT_SPEC_PROFILE> -bb_list <LIST_PDB_COMPLEXES> -dump_transfac <PATH_TO_OUTPUT_TRANSFAC> -resfile <RESFILE> -nooutput true –score:weights talaris2013 @<PATH_TO_FLAGS>

**Outputs**

Log contains probabilities per rotamer, probabilities per amino acid, and distances from experimental specificity profile (if provided). If <PATH_TO_OUTPUT_TRANSFAC> is provided, dumps one transfac file per backbone, file with backbone Boltzmann probabilities, and one averaged transfac file for the ensemble as a whole.

**Distances.py**

**Inputs**

1. **Transfac file as output by MFPred**
2. **Experimental specificity profile**

**Process**

import os

import sys

import numpy as np

import math

from sklearn import metrics

import matplotlib.pyplot as plt

from pylab import *

def binarizeList ( firstList ):

    binary_freq = []

    choose_val = 0.10

    max_val = max(firstList)

    if max_val < 0.10:

        if max_val > 0.09:

          choose_val = 0.09

        elif max_val > 0.08:

            choose_val = 0.08

        elif max_val > 0.07:

            choose_val = 0.07

    for val in firstList:

        if val > choose_val:

            binary_freq.append( 1 )

        else:

            binary_freq.append( 0 )

    return binary_freq

def areaUnderCurve ( firstList, secondList ):

    binary_freq = binarizeList( firstList )

    fpr, tpr, _ = metrics.roc_curve(binary_freq, secondList)

    auc = metrics.auc(fpr,tpr)

    return auc

def shannonEntropy( firstList ):

    sE = -1.0 * np.sum( [ p * math.log(p,2) for p in firstList if p != 0.0 ] )

    return sE

def JSDivergence( firstList, secondList ):

    firstSE = shannonEntropy( firstList )

    secondSE = shannonEntropy( secondList )

    combList = [ 0.5 * fL + 0.5 * sL for fL,sL in zip(firstList, secondList) ]

    combSE = shannonEntropy( combList )

    return combSE - 0.5 * firstSE - 0.5 * secondSE

def cosineDist( firstList, secondList):

    dotP = np.dot(firstList, secondList)

    sqrt_1 = math.sqrt( np.sum( np.power( firstList,2 ) ) )

    sqrt_2 = math.sqrt( np.sum( np.power( secondList,2 ) ) )

    return dotP/(sqrt_1 * sqrt_2)

def frobDist( firstList, secondList):

    diff_lists = np.subtract(firstList,secondList)

    terms = np.power( diff_lists,2)

    return math.sqrt( np.sum( terms ) )

def aveAbsDist( firstList, secondList ):

    diff_lists = np.fabs( np.subtract( firstList, secondList) )

    return sum( diff_lists ) / len( diff_lists )

def readSpecProfileList( filename ):

    with open(filename) as transfac_file:

        transfac = transfac_file.readlines()

    motifWidth = len(transfac)-2

    aaAlpha = transfac[1].split()[1:]

    freq = [{k: 0.0 for k in aaAlpha} for i in range(motifWidth)]

    t_read = transfac[2:]

    for pos,line in enumerate( t_read,0 ):

        for aa_ind,f in enumerate( line.split()[1:], 0):

            freq[pos][aaAlpha[aa_ind]] = float(f)

    freqList = [ [ val for key,val in sorted(pos.iteritems()) ] for pos in freq ]

    return freqList

def main(args):

    infile = args[1]

    infile_expt = args[2]

    expt = os.path.basename(infile_expt).rstrip()

    expt = expt.rsplit('.',1)[0]

    tokens=infile.rsplit('.',1)

    file=tokens[0]

    outfile= '%s_dist.txt' % (file)

    outfile_heat= '%s_heat.png' % (file)

    freq_in = readSpecProfileList( infile )

    freq_expt = readSpecProfileList( infile_expt )

    nda_freq_in = np.array( [ freq_in] )

    nda_freq_expt = np.array( [ freq_expt] )

    flat_freq_in = np.ndarray.flatten( nda_freq_in )

    flat_freq_expt = np.ndarray.flatten( nda_freq_expt )

    c = [ cosineDist( i, g ) for i,g in zip( freq_in, freq_expt ) ]

    f = [ frobDist( i, g ) for i,g in zip( freq_in, freq_expt ) ]

    a = [ aveAbsDist( i, g ) for i,g in zip( freq_in, freq_expt ) ]

    jsd1 = [ JSDivergence ( i, g ) for i,g in zip( freq_in, freq_expt )]

    auc = [ areaUnderCurve ( i, g ) for i, g in zip( freq_expt, freq_in )]

    avg_c = cosineDist( flat_freq_in, flat_freq_expt )

    avg_f = frobDist( flat_freq_in, flat_freq_expt )

    avg_a = aveAbsDist( flat_freq_in, flat_freq_expt )

    avg_jsd = np.sum(jsd1) / len(jsd1)

    avg_auc = np.sum(auc) / len(auc)

    c.append(avg_c)

    f.append(avg_f)

    a.append(avg_a)

    jsd1.append(avg_jsd)

    auc.append(avg_auc)

    dist_out = open(outfile,"w")

    dist_out.write("Metric\t")

    dist_out.write("\t".join([ "Col{0}".format(i) for i in xrange(1,len(c)) ]))

    dist_out.write("\tAvg\nCosine\t")

    dist_out.write("\t".join(map(str,c)))

    dist_out.write("\nFrobenius\t")

    dist_out.write("\t".join(map(str,f)))

    dist_out.write("\nAAD\t")

    dist_out.write("\t".join(map(str,a)))

    dist_out.write("\nJSD\t")

    dist_out.write("\t".join(map(str,jsd1)))

    dist_out.write("\nAUC\t")

    dist_out.write("\t".join(map(str,auc)))

    dist_out.write("\n")

    dist_out.close()

if __name__ == "__main__":

  main(sys.argv)

**Outputs**

Distances file: the name of this file is <INPUT_FILE>_dist.txt. Contains one line per metric. Each line contains one value per column and the last value is the average of the columns.

**Non-MFPred pipeline software – used for controls and/or optimization of protocol:**

**Backbone Ensemble Generation**

**Thread Peptide Alone (pre-flexpepdock or pre-backrub)**

**Command Line:**

<ROSETTA_BIN>rosetta_scripts.static.linuxgccrelease -nstruct 1 -jd2:ntrials 1 -parser:protocol <XML_FILE> -database <ROSETTA_DB> <CONST_ARG> -s <STARTING_RELAXED_MODEL> -run:preserve_header -overwrite @<PATH_TO_FLAGS>

**Sample xml:**

<ROSETTASCRIPTS>

        <SCOREFXNS/>

        <TASKOPERATIONS>

                <InitializeFromCommandline name="init"/>

                <ReadResfile filename=”<RESFILE>” name="rrf"/>

        </TASKOPERATIONS>

        <FILTERS/>

        <MOVERS>

<MutateResidue name="mut1" target="<PEPT_RES1>" new_res="DM1"/>

<MutateResidue name="mut2" target="<PEPT_RES2>" new_res="DM2"/>

<MutateResidue name="mut3" target="<PEPT_RES3>" new_res="DM3"/>

<MutateResidue name="mut4" target="<PEPT_RES4>" new_res="DM4"/>

<MutateResidue name="mut5" target="<PEPT_RES5>" new_res="DM5"/>

<MutateResidue name="mut6" target="<PEPT_RES6>" new_res="DM6"/>

<MutateResidue name="mut7" target="<PEPT_RES7>" new_res="DM7"/>

              <AddOrRemoveMatchCsts cst_instruction="add_new" name="cstadd"/>

              <PackRotamersMover name="repack" task_operations="rrf,init"/>

        </MOVERS>

        <APPLY_TO_POSE/>

        <PROTOCOLS>

                <Add mover_name="mut1"/>

                <Add mover_name="mut2"/>

                <Add mover_name="mut3"/>

                <Add mover_name="mut4"/>

                <Add mover_name="mut5"/>

                <Add mover_name="mut6"/>

                <Add mover_name="mut7"/>

                <Add mover_name="cstadd"/>

                <Add mover_name="repack"/>

        </PROTOCOLS>

</ROSETTASCRIPTS>

**Resfile:**

NATRO all, NATAA peptide residues

**Flags:**

-mute core.io.database

-packing::use_input_sc

-packing::extrachi_cutoff 1

-packing::ex1

-packing::ex2

-linmem_ig 10

-out:file::output_virtual

**FlexPepDock**

**Command line:**

<ROSETTA_BIN>_scripts.static.linuxgccrelease -parser:protocol ~/mean_field/xml/flexpepdock.xml -database <ROSETTA_DB> -s <STARTING_THREADED_MODEL> -ex1 -ex2 -ex1aro -ex2aro -extrachi_cutoff 0 -nstruct 10 -enzdes:cstfile <PATH_TO_ENZDES_CSTFILE> -score:weights talaris2013_cst -run:preserve_header -packing:use_input_sc

**Sample xml:**

<ROSETTASCRIPTS>

        <TASKOPERATIONS>

        </TASKOPERATIONS>

        <SCOREFXNS>

        </SCOREFXNS>

        <FILTERS>

        </FILTERS>

        <MOVERS>

                <AddOrRemoveMatchCsts name="cstadd" cst_instruction="add_new" />

                <FlexPepDock name="fpd" pep_refine="1" />

        </MOVERS>

        <APPLY_TO_POSE>

        </APPLY_TO_POSE>

        <PROTOCOLS>

                <Add mover_name="cstadd"/>

                <Add mover_name="fpd"/>

        </PROTOCOLS>

</ROSETTASCRIPTS>

**Backrub**

**Command line:**

<ROSETTA_BIN>backrub_cst.linuxgccrelease -run:preserve_header -score:weights talaris2013_cst -database <ROSETTA_DB> -s <STARTING_THREADED_MODEL> -ex1 -ex2 -ex1aro -ex2aro -extrachi_cutoff 0 -backrub:minimize_movemap <MOVEMAP_FILE> -backrub:ntrials 10000 -backrub:pivot_residues 215 216 217 218 219 220 221 222 223 224 -overwrite -enzdes:cstfile <PATH_TO_ENZDES_CSTFILE> -packing:use_input_sc

**Movemap:**

RESIDUE * CHI

JUMP * YES

CHAIN 2 BBCHI

**Backrub_cst app:**

This app is a version of the general backrub app that includes Enzdes style constraint as a mover. Currently, the general backrub app has been moved to a new Mover called BackrubProtocol mover – had this been available at the time of benchmarking, this would have been used instead.

**Enumerate_dihedral**

**Command line:**

<ROSETTA_BIN>enumerate_dihedral.linuxgccrelease -database <ROSETTA_DB> -s <STARTING_RELAXED_MODEL> -anchor_res <FIXED_RES_P1> -run:preserve_header

**Enumerate dihedral app:**

This app is in my pilot apps folder within the Rosetta source code (Rosetta/main/source/src/apps/pilot/arubenstein/enumerate_dihedral.cc).

**Clustering via AmberTools cpptraj:**

**Run tleap to convert pdb to topology and coordinate files:**

**tleap.in:**

source leaprc.ff14SB

source leaprc.phosaa10

loadAmberParams frcmod.ionsjc_tip3p

pdb = loadpdb <PDB_NAME>

addions pdb Cl- 0

addions pdb Na+ 0

#solvatebox pdb TIP3PBOX 10.0

saveamberparm pdb <PDB_NAME>.top <PDB_NAME>.crd

**Run:**

tleap -f tleap.in

**Run cpptraj to cluster:**

**cpptraj.in file:**

parm <TOPO_FILE_1>

trajin <COORD_FILE_1>

parm <TOPO_FILE_2>

trajin <COORD_FILE_2>

.

.

.

cluster hieragglo clusters <N_CLUSTERS> rms :<PEPT_BEG_RES>-<PEPT_END_RES> repout <N_CLUSTERS> repfmt pdb

**Run:**

cpptraj -i 'cpptraj.in'

**Multispecificity Prediction Controls for MFPred**

**Monte-Carlo (pepspec)**

**Command-line:**

<ROSETTA_BIN>mc_no_sa.linuxgccrelease -database <ROSETTA_DB> -pepspec:pdb_list <BACKBONE_ENSEMBLE_LIST> -save_low_pdbs false -pepspec:n_peptides 1 -pepspec:use_input_bb true -ex1 -ex2 -extrachi_cutoff 0 -pepspec:diversify_lvl 50 -pepspec:run_sequential -use_input_sc

**Mc_no_sa app:**

This app is a version of the general pepspec app that includes profiling (necessary to extract running times and determine speedup).

**Genetic Algorithm (sequence_tolerance)**

**Command-line:**

<ROSETTA_BIN>sequence_tolerance_control.linuxgccrelease -database <ROSETTA_DB> -s <MODEL_FROM_BACKBONE_ENSEMBLE> -ex1 -ex2 -ex1aro -ex2aro -extrachi_cutoff 0 -ms:generations 5 -ms:pop_size 2000 -ms:pop_from_ss 1 -ms:checkpoint:prefix <PREFIX> -ms:checkpoint:interval 200 -ms:checkpoint:gz -seq_tol:fitness_master_weights 1 1 1 2 -resfile <RESFILE>

**Resfile:**

NATAA residues according to seqtol_resfile.py, ALLAA peptide residues

**Sequence_tolerance_control app:**

This app is a version of the general sequence_tolerance app that includes profiling (necessary to extract running times and determine speedup).
